# Distinct lymphocyte antigens 6 (Ly6) family members Ly6D, Ly6E, Ly6K and Ly6H drive tumorigenesis and clinical outcome

**DOI:** 10.18632/oncotarget.7163

**Published:** 2016-02-03

**Authors:** Linlin Luo, Peter McGarvey, Subha Madhavan, Rakesh Kumar, Yuriy Gusev, Geeta Upadhyay

**Affiliations:** ^1^ Innovation Center for Biomedical Informatics (ICBI), Georgetown University Medical Center, Washington, District of Columbia 20007, United States of America; ^2^ Department of Biochemistry and Molecular Medicine, School of Medicine and Health Sciences, George Washington University, Washington, District of Columbia 20037, United States of America; ^3^ Department of Oncology, Lombardi Comprehensive Cancer Center, Georgetown University Medical Center, Washington, District of Columbia 20007, United States of America

**Keywords:** cancer biomarkers, stem cell genes, poor prognosis, lymphocyte antigens 6 complex, Ly6 genes

## Abstract

Stem cell antigen-1 (Sca-1) is used to isolate and characterize tumor initiating cell populations from tumors of various murine models [1]. Sca-1 induced disruption of TGF-β signaling is required *in vivo* tumorigenesis in breast cancer models [2, 3-5]. The role of human Ly6 gene family is only beginning to be appreciated in recent literature [6-9]. To study the significance of Ly6 gene family members, we have visualized one hundred thirty gene expression omnibus (GEO) dataset using Oncomine (Invitrogen) and Georgetown Database of Cancer (G-DOC). This analysis showed that four different members Ly6D, Ly6E, Ly6H or Ly6K have increased gene expressed in bladder, brain and CNS, breast, colorectal, cervical, ovarian, lung, head and neck, pancreatic and prostate cancer than their normal counter part tissues. Increased expression of Ly6D, Ly6E, Ly6H or Ly6K was observed in sub-set of cancer type. The increased expression of Ly6D, Ly6E, Ly6H and Ly6K was found to be associated with poor outcome in ovarian, colorectal, gastric, breast, lung, bladder or brain and CNS as observed by KM plotter and PROGgeneV2 platform. The remarkable findings of increased expression of Ly6 family members and its positive correlation with poor outcome on patient survival in multiple cancer type indicate that Ly6 family members Ly6D, Ly6E, Ly6K and Ly6H will be an important targets in clinical practice as marker of poor prognosis and for developing novel therapeutics in multiple cancer type.

## INTRODUCTION

The lymphocyte antigen-6 (Ly6) complex, a group of alloantigens, was first discovered in mice approximately 40 years ago on lymphocytes [3, 4]. Ly6 family members are evolutionary conserved and have been mapped to human chromosome 8, in particular, the 8q24.3 locus, which is syntenic to murine chromosome 15 [9, 10]. The founding Ly6 member CD59 was identified in human lymphoid cells with a role in the complement membrane attack complex and T cell activation [11]. To date, 20 human Ly6 proteins, ranging from 11-36 kDa, have been identified and categorized as either transmembrane or secretory based on the availability of a GPI-anchored signal sequence [9]. Ly6 family is located on chromosome 8q24 alongside c-Myc. The somatic copy number gain in 8q has been associated with most prevalent copy number gain in multiple cancer types [12, 13]. Ly6E and Ly6K has been implicated in development of novel therapeutics in multiple cancers [7, 8, 14, 15]. We have previously shown that increased levels of Ly6A/E (Sca-1) promote breast tumorigenesis via disruption of TGF-β signaling and suppression of GDF10 expression in mouse models [2]. GDF10 has been shown to regulate epithelial to mesenchymal transition, growth and invasion in oral squamous cell carcinoma [16]. These finding suggest that Ly6 genes family members have important role multiple cancer but a comprehensive analysis of multiple members of Ly6 gene family and its relation to cancer patient survival is lacking.

Here we evaluate the importance and significance of novel Ly6 family in cancer prognosis and treatment using publically available datasets of gene expression micro array analysis coupled with clinical outcome information. To study the status of Ly6D, Ly6E, Ly6H and Ly6K mRNAs in human normal and cancer tissues in one-hundred and thirty gene expression omnibus (GEO) dataset using Oncomine (Invitrogen) or Georgetown Database of Cancer (G-DOC). The expression status of Ly6D, Ly6E, Ly6H and Ly6K in caner tissue was correlated with patient outcome using KM plotter and PROGgeneV2 platform.

## RESULTS

### Increased expression of Ly6D in multiple cancers

To examine the status of Ly6D in human cancer, we used Oncomine or G-DOC to analyze gene expression omnibus (GEO) datasets. The data summarized in Table 1 showed a significant increased expression of Ly6D in bladder cancer (n=150) than normal tissues (n=57) in Sanchez-Carbayo [17] and Dryskjot [18] studies. Ly6D mRNA expression was increased significantly in brain cancer (n=131) than normal tissues (n=23) in Sun study [19]. Ly6D mRNA expression was increased significantly in breast cancer (n=1597) than normal tissues (n=153) in Curtis study [20] and Lin study [21]. Ly6D mRNA expression was increased significantly in head and neck cancer (n=56) than normal tissues (n=41) in Estilo [22], He [23] and Frierson [24] studies. Ly6D mRNA expression was increased significantly in gastric cancer (n=31) than normal tissues (n=19) in Cho [25] study. Ly6D mRNA expression was increased significantly in lung cancer (n=453) than normal tissues (n=244) in Landi [26], Selamat [27], Su [28], Okayana [29], Bhattacharjee [30], Hou [31], Wachi [32] studies. Ly6D mRNA expression was increased significantly in ovarian cancer (n=221) than normal tissues (n=18) in Wachi [32], Welsh [33], Hendrix [34] and Bonome [35] studies. Ly6D mRNA expression was increased significantly in pancreatic cancer (n=75) than normal tissues (n=55) in Pei [36] and Badea [37] studies. Ly6D mRNA expression was increased significantly in colorectal cancer (n=369) than normal tissues (n=150) in The Cancer Genome Atlas (TCGA), Sabates-Bellver [38], Kaiser [39], Gaedcke [40] and Skrzypczak [41] studies. Ly6D mRNA was increased significantly in Kidney cancer (n=53) than normal tissues (n=28) in Jones [42] and Yusenko [43] studies.

**Table 1:** Ly6D mRNA expression in normal and tumor tissue (n=number of samples) of multiple cancer types

Type of cancer	Reference	N (Normal)	N (Cancer)	Fold change	P-value
**Bladder**	[17]	48	28 (Superficial)	39.50	3.5E-12
			81 (Infiltrating)	6.63	2.6E-06
	[18]	9	28 (Superficial)	3.30	5.5E-04
			13 (Infiltrating)	3.17	8.0E-03
**Brain and CNS**	[19]	23	50 (Oligodendroglia)	1.27	1.3E-02
			81 (Glioblastoma)	1.20	3.6E-02
**Breast**	[20]	144	32 (Medullary)	1.58	2.0E-03
			1556 (Invasive ductal)	1.03	5.0E-03
	[21]	9	9 (Cancer)	1.21	1.5E-02
**Head and neck**	[22]	26 (Tongue)	31 (Tongue squamous)	7.56	4.5E-05
	[23]	9 (Thyroid)	9 (Thyroid papillary)	1.23	4.0E-03
	[24]	6 (Salivary)	16 (Salivary adenoid)	4.56	2.6E-02
**Gastric**	[25]	19	31 (Adeno)	1.25	8.0E-03
**Lung**	[26]	49	58 (Adeno)	1.43	5.1E-04
	[27]	58	58 (Adeno)	1.11	8.0E-03
	[28]	30	27 (Adeno)	2.29	1.0E-03
	[29]	20	226 (Adeno)	3.52	2.2E-07
	[30]	17	21 (Squamous)	12.71	2.0E-03
	[31]	65	27 (Squamous)	7.18	2.7E-06
	[32]	5	5 (Squamous)	3.28	3.7E-02
**Ovarian**	[33]	4	28 (Papillary)	1.07	1.0E-06
	[34]	4	8 (Clear cell adeno)	1.37	2.7E-02
	[35]	10	185 (Cancer)	1.17	8.4E-04
**Pancreatic**	[36]	16	36 (Cancer)	3.98	2.7E-06
	[37]	39	39 (Ductal adeno)	1.50	2.9E-02
**Colorectal**	TCGA	19	29 (Colon Mucinary adeno)	5.88	2.2E-07
		3	24 (Cecum adeno)	3.35	3.2E-07
		3	6 (Rectal Mucinary adeno)	3.22	1.1E-02
		3	60 (Rectal adeno)	2.78	9.4E-08
		19	102 (Colon adeno)	2.15	2.4E-12
	[38]	9	25 (Colon adeno)	2.25	4.0E-03
	[39]	5	13 (Colon Mucinary adeno)	1.62	5.0E-03
	[40]	65	65 (Rectal adeno)	1.57	1.9E-07
	[41]	24	45 (Colon adeno)	1.33	5.2E-04
**Kidney**	[42]	23	8 (Cancer)	4.22	2.9E-02
	[43]	5	19 (Papillary)	2.49	3.0E-03
			26 (Clear cell)	1.92	1.2E-02

Ly6D mRNA expression was significantly increased in bladder, brain and CNS, breast, head and neck, gastric, lung, ovarian, pancreatic, colorectal, and kidney cancer than their normal counterpart. Data observed using Oncomine (Invitrogen). N=number of patient samples.

These results show that Ly6D expression was significantly increased in bladder, brain and CNS, breast, head and neck, gastric, lung, ovarian, pancreatic, colorectal and kidney cancer than their counterpart normal tissues.

The data summarized in Table 2 showed that Ly6D mRNA expression was increased significantly in subtypes of multiple cancers. Ly6D mRNA expression was significantly higher in superficial bladder cancer (n=179) than infiltrating bladder cancer (n=175) in Sanchez-Carbayo [17], Stransky [44] and Lee [45] studies. Ly6D mRNA expression was significantly higher in medulloblastoma (n=60) than rhabdoid tumor (n=5) Pomeroy [46] study. Ly6D mRNA expression was significantly increased in triple negative breast cancer (TNBC) (n=700) compared to 2667 sample of non-TNBC (n=2667), grade 3 (n=47) than grade 2 (n=27), grade N1 (n=190) than grade N0 (n=137), tumors with p53 mutation (n=130) than p53 wildtype tumors (n=261), tumors with BRCA1 mutation (n= 38) than BRCA1 wildtype tumors (n=157), ERBB2 positive (n=92) than ERBB2 negative (n=48) tumors, basal type (n=16) than luminal (n=27) in The Cancer Genome Atlas (TCGA) (Unpublished, NCI), Stickeler [47], Minn [48], Waddell [49], Gluck [50], Bild [51], Kao [52], Bittner (unpublished, GSE2109), Farmer [53], Korde [54], Richardson [55], Esserman [56], Chin [57], Ginestier [58], vantVeer [59], Curtis [60], Ivshina [61], Bonnefoi [62] and Hatzis [63] studies. Ly6D mRNA expression was significantly upregulated in microsatellite instability in gastric and colorectal cancer as seen by D'Errico [64] and Jorissen [65] studies. High Ly6D mRNA expression was also correlated in more aggressive subsets of cervical cancer, esophageal and kidney cancer in Bittner (unpublished, GSE2109), Pyeon [66], TCGA [67], Kimchi [68] studies. Interestingly, in case of breast cancer depicted in Table 2, 14 studies show that Ly6D is significantly increased in TNBC while only one study show it is higher in ERBB2+ cancer compared to ERBB2− tumors. This suggests that while Ly6D is predominantly associated with TNBC tumor.

**Table 2:** Ly6D mRNA expression in subset of multiple cancers

Type of cancer	Reference	Cancer		Fold change	P-value
		N (Group 1)	N (Group 2)		
**Bladder**	[17]	81 (Infiltrating)	28 (Superficial cancer)	5.96	7.6E-05
	[44]	32 (Infiltrating)	25 (Superficial cancer)	5.96	7.6E-05
	[45]	62 (Infiltrating)	126 (Superficial cancer)	1.75	8.0E-03
**Brain and CNS**	[46]	5 (Rhabdoid Tumor)	60 (Medulloblastoma)	24.87	4.0E-03
		40 (Medullo Alive at 1 year)	6 (Dead at 1 year)	3.18	5.9E-04
	[69]	60 (Astrocytoma Alive at 1 year)	16 (Dead at 1 year)	1.27	3.7E-01
**Pancreatic**	[70]	5 (Alive at 3 year))	19 (Dead at 3 year)	1.46	9.0E-03
**Breast**	TCGA	250 (Non-TNBC)	46 (TNBC)	5.42	3.1E-12
	[47]	24 (Non-TNBC)	8 (TNBC)	8.92	1.2E-02
		15 (Grade 2)	16 (Grade 3)	4.08	3.2E-02
	[48]	69 (Metastasis free 3 year)	12 (Metastasis at 3 year)	2.91	3.5E-02
		71 (Non-TNBC)	25 (TNBC)	2.04	1.2E-02
	[49]	44 (Non-TNBC)	22 (TNBC)	3.36	8.4E-04
		60 (BRCA1 wildtype)	20 (BRCA1 Mutation)	2.02	2.5E-02
	[50]	101 (Non-TNBC)	50 (TNBC)	3.22	1.0E-08
		72 (TP53 wildtype)	72 (TP53 Mutation)	1.63	6.0E-03
	[51]	124 (Alive at 3 year)	27 (Dead at 3 year)	1.90	3.8E-02
		60 (Alive at 5 year)	42 (Dead at 5 year)	1.62	3.4E-02
		48 (ERBB2 neg)	92 (ERBB2 pos)	1.51	2.0E-02
	[52]	295 (Non-TNBC)	32 (TNBC)	3.01	9.7E-04
		16 (Metastasis free 3 year)	67 (Metatasis at 3 year)	1.99	3.9E-02
		295 (Alive at 3 year)	31(Dead at 3 year)	2.74	9.7E-04
		137 (Grade N0)	190 (Grade N1+)	1.48	6.0E-03
	[71]	14 (Metastasis free 5 year)	172 (Metastasis at 5 year)	2.91	1.6E-04
	GSE2109	129 (Non-TNBC)	39 (TNBC)	1.79	2.4E-02
	[53]	27 (Luminal)	16 (Basal)	2.47	1.0E-03
	[54]	39 (Non-TNBC)	21 (TNBC)	2.28	3.9E-04
	[55]	19 (Non-TNBC)	18 (TNBC)	2.19	1.2E-02
	[56]	74 (Non-TNBC)	24 (TNBC)	2.10	4.8E-04
		77 (Alive at 3 year)	15(Dead at 3 year)	2.74	9.7E-04
	[57]	87 (Non-TNBC)	19 (TNBC)	1.88	6.0E-03
	[58]	12 (Grade 2)	31 (Grade 3)	1.81	1.6E-02
	[59]	97 (BRCA1 wildtype)	18 (BRCA1 Mutation)	1.64	6.7E-04
	[60]	1340 (Non-TNBC)	211 (TNBC)	1.43	2.0E-03
	[61]	189 (TP53 Wildtype)	58 (TP53 (Mutation)	1.49	3.4E-04
	[62]	32 (Non-TNBC)	80 (TNBC)	1.43	8.6E-06
	[63]	320 (Non-TNBC)	178 (TNBC)	1.41	7.6E-09
**Gastric**	[64]	12 (Microsatellite stable)	14 (Microsatellite Instable)	3.94	6.0E-03
**Cervical**	GSE2109	9 (Adeno)	23 (Squamous)	12.05	2.0E-03
	[66]	15 (Stage M0)	4 (Stage M1+)	3.20	1.1E-02
**Colorectal**	TCGA [67]	24 (Cecum adeno)	20 (Colon Muc adeno)	1.89	6.0E-03
	[65]	16 (rectal adeno)	137 (Colon adeno)	1.75	1.2E-02
		77 (Microsatellite stable)	78 (Microsatellite unstable)	1.45	2.9E-02
	[72]	15 (Alive at 5 year)	20 (Dead at 5 year)	1.42	3.9E-02
**Esophageal**	[68]	8 (Precursor)	8 (Cancer)	46.55	4.0E-03
**Kidney**	TCGA [67]	72 (Clear cell)	16 (Papillary)	4.12	4.0E-03
	GSE2109	10 (Grade 2)	6 (Grade 3)	2.02	1.5E-02

Ly6D mRNA expression was significantly increased in subtypes of bladder, brain and CNS, pancreatic, breast, gastric, cervical, colorectal, esophageal and kidney cancer. High Ly6D expression was significantly correlated with poor clinical outcome in brain and CNS, pancreatic, and colorectal cancer. Data observed using Oncomine (Invitrogen). N=number of patient samples.

These results show that Ly6D expression was significantly increased in subtypes of bladder, brain and CNS, breast, pancreatic, gastric, cervical, colorectal, esophageal and kidney cancer.

### High Ly6D expression and survival outcome in multiple cancers

Table 2 also shows that high Ly6D mRNA expression in brain cancer was significantly correlated with decreased one-year survival (dead, n=22 vs alive, n=100) in Pomeroy [46] and Phillips [69] studies. High Ly6D mRNA expression in pancreatic cancer was significantly correlated with decreased three-year survival (dead, n=19 vs alive, n=5) in Collisson [70] study. High Ly6D mRNA expression in breast cancer was significantly correlated with decreased three-year metastasis free survival (metastasis, n=79 vs metastasis free, n=85), decreased five-year metastasis free survival (metastasis, n=172 vs metastasis free, n=14), decreased three-year survival (dead, n=73 vs alive, n=496) decreased five-year survival (dead, n=42 vs alive, n=60) in Minn [48], Bild [51], Kao [52], Bos [71], and Essermann [56] studies.

**Figure 1:**
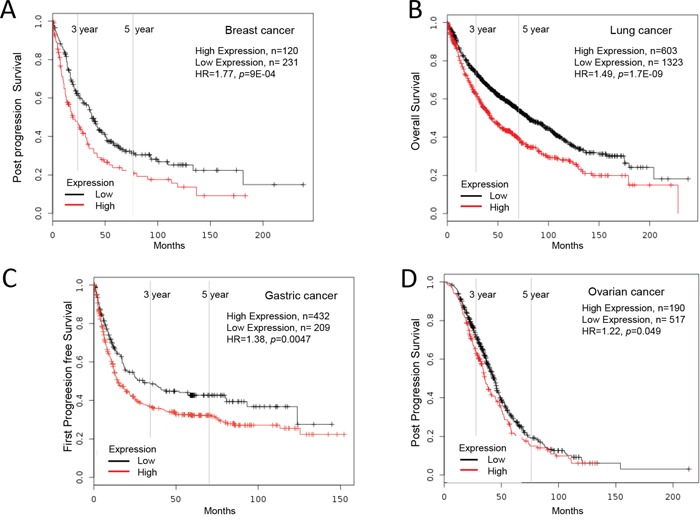
Increased Ly6D mRNA expression in cancer and patient survival High Ly6D expression leads to poor survival in **A.** breast cancer, **B.** lung cancer, **C.** gastric cancer and **D.** ovarian cancer.

High Ly6D mRNA expression in breast cancer was significantly correlated with poor outcome in five-year distant metastasis free survival (low Ly6D, n=818; high Ly6D, n=790; HR=1.29, *p*=0.012, n= number of patient, HR=hazard ratio), post progression free survival (low Ly6D, n=231; high Ly6D, n=120; HR=1.57, *p*=9.0E-04), relapse free survival (low Ly6D, n=1133; high Ly6D, n=2421; HR=1.30, *p*=2.0E-05) shown by KM plotter and five-year relapse free survival (low Ly6D, n=57; high Ly6D, n=57; HR=1.48, *p*=0.006) shown by PROGgeneV2 (Table S1, Figure 1A).

High Ly6D mRNA expression in colon cancer was significantly correlated with poor outcome in relapse free survival (low Ly6D, n=25; high Ly6D, n=26; HR=1.19, *p*=0.0469) and overall survival (low Ly6D, n=25; high Ly6D, n=26; HR=1.63, *p*=0.0199) shown by PROGgeneV2 (Table S1) and overall survival in 5-year overall survival (low Ly6D, n=15; high Ly6D, n=20, p=3.9E-02) in Smith study [72] (Table 2).

High Ly6D mRNA expression in lung cancer was significantly correlated with poor outcome in five-year overall survival with no restriction (low Ly6D, n=1323; high Ly6D, n=603; HR=1.49, *p*=1.70E-09) or with restriction of lung adenocarcinoma (low Ly6D, n=538; high Ly6D, n=181; HR=2.11, *p*=7.60E-10), first progression free survival with no restriction (low Ly6D, n=712; high Ly6D, n=270; HR=1.33, *p*=0.006) or with restriction of lung adenocarcinoma (low Ly6D, n=345; high Ly6D, n=116; HR=1.71, *p*=0.001) and post progression free survival with restriction of lung adenocarcinoma (low Ly6D, n=257; high Ly6D, n=87; HR=1.48, *p*=0.006), by KM plotter and five-year relapse free survival with restriction of lung adenocarcinoma (low Ly6D, n=112; high Ly6D, n=113; HR=1.38, *p*=4.00E-04) shown by PROGgeneV2 (Table S1, Figure 1B).

High Ly6D mRNA expression in gastric cancer was significantly correlated with poor outcome in five-year post progression free survival (low Ly6D, n=209; high Ly6D, n=432; HR=1.38, *p*=0.0047) shown by KM plotter (Table S1, Figure 1C).

High Ly6D mRNA expression in ovarian cancer was significantly correlated with poor outcome in five-year post progression free survival (low Ly6D, n=517; high Ly6D, n=190; HR=1.22, *p*=0.049) shown by KM plotter (Table S1, Figure 1D).

These data show that high Ly6D expression was significantly correlated with poor clinical outcome in brain and CNS, pancreatic, and colorectal, breast, colorectal, lung, gastric and ovarian cancer.

### Increased expression of Ly6E in multiple cancers

To examine the status of Ly6E in human cancer, we used Oncomine or G-DOC to analyze gene expression omnibus (GEO) datasets. The data in Table 3A shows a significant increased expression of Ly6E in bladder cancer (n=150) than normal tissues (n=57) in Sanchez-Carbayo [17] and Dryskjot [18] studies. Ly6E mRNA expression was significantly increased in breast cancer (n=2613) than normal tissues (n=235) in TCGA, Radvani [73], Curtis [20], Ma [74], Gluck [75], and Zhao [76] studies. Ly6E mRNA expression was significantly increased in esophageal cancer (n=78) than normal tissues (n=78) in Kimchi [68], Hu [77] and Su [78] studies. Ly6E mRNA expression was significantly increased in gastric cancer (n=89) than normal tissues (n=62) in D'Errico [64], Cho [25] and Wang [79] studies. Ly6E mRNA expression was significantly increased in pancreatic cancer (n=85) than normal tissues (n=60) in Logsdon [80], Badea [37] and Pei [81] studies. Ly6E mRNA expression was significantly increased in cervical cancer (n=90) than normal tissues (n=34) in Scotto [82], Pyeon [66] and Biewenga [83] studies. Ly6E mRNA expression was significantly increased in colorectal cancer (n=10) than normal tissues (n=5) in Skrzypczak [84] study. Ly6E mRNA expression was significantly increased in prostate cancer (n=36) than normal tissues (n=17) in Tomlins [85] study. The data in Table 3B shows a significantly increased Ly6E mRNA expression in lung cancer (n=514) than normal tissues (n=220) in Okayama [29], Talbot [86], Beer [87], Su [28], Wei [88], Selamat [27] and Landi [26] studies. Ly6E mRNA expression was significantly increased in head and neck cancer (n=396) than normal tissues (n=17) Toruner [89], Giordano [90], Ye [91], Peng [92], Peng 2 [92], He [23], Cromer [93], Estilo [22], Vasko [94], Ginos [95] and Frierson [24] studies. Ly6E mRNA expression was significantly increased in ovarian cancer (n=396) than normal tissues (n=40) in Yoshihara [96], Adib [97], TCGA (NCI, unpublished), Welsh [98], Bonome [99] and Henedrix [34] studies. Ly6E mRNA expression was significantly increased in kidney cancer (n=155) than normal tissues (n=68) in Yusenko [43], Beroukhim [100], Jones [42], Cutcliffe [101], Gumz [102], and Lenburg [103] studies. Ly6E mRNA expression was significantly increased in melanoma (n=45) than normal skin (n=7) in Talantov [104] study. Ly6E mRNA expression was significantly increased in embryonic tumors (n=24) than normal testis (n=6) in Korkola [105] study and pleural malignant mesothelioma (n=40) than normal samples (n=9) of pleura in Gordon [106] study.

Table 3Ly6E mRNA expression in normal and tumor tissue of multiple cancer types

**Table:** A. Ly6E is significantly increased in bladder, breast, Esophageal, gastric, pancreatic, cervical, colorectal and prostate cancer than their normal counterparts

Type of cancer	Reference	N (Normal)	N (Cancer)	Fold change	P-value
**Bladder**	[17]	48	28 (Superficial)	4.64	4.3E-12
			81 (Infiltrating)	3.09	2.1E-08
	[18]	9	13 (Infiltrating)	2.09	4.7E-04
			28 (Syperficial)	1.70	1.8E-04
**Breast**	TCGA	61	3 (Adeno)	1.42	1.5E-02
			76 (Invasive)	1.82	2.4E-08
			389 (Invasive Ductal)	1.47	1.7E-06
			36 (Invasive Lobular)	1.32	1.3E-02
	[73]	9	3 (Ductal)	3.25	3.0E-03
			3 (Invasive Medulary)	2.17	1.5E-02
	[20]	144	32 (Medullary)	2.10	2.9E-05
			1556 (Invasive Ductal)	1.55	4.8E-21
			46 (Mucinous)	1.65	3.0E-05
			10 (Ductal)	1.42	2.1E-02
			21(Invasive)	1.35	1.6E-02
			148 (Invasive Lobular)	1.15	8.0E-03
			90 (Invasive Lobular & Ductal)	1.13	4.9E-02
	[74]	14	9 (Ductal)	1.18	4.0E-03
	[75]	4	154 (Invasive)	1.19	6.0E-03
	[76]	3	37 (Invasive Ductal)	1.91	3.6E-02
**Esophageal**	[68]	8	8 (Adeno)	2.21	2.4E-02
	[77]	17	17 (Squamous)	3.00	4.3E-05
	[78]	53	53 (Squamous)	1.78	7.7E-13
**Gastric**	[64]	31	26 (Intestinal Adeno)	9.65	2.3E-12
	[25]	19	31 (Diffuse Aden)	3.40	3.5E-07
			20 (Intestinal Adeno)	2.94	2.9E-04
	[79]	12	12 (Cancer)	2.46	5.0E-03
**Pancreatic**	[80]	5	10 (Adeno)	3.21	5.3E-04
	[37]	39	39 (PD adeno)	3.05	5.2E-16
	[81]	16	36 (Tumor)	3.49	2.1E-07
**Cervical**	[82]	21	32 (Squamous)	2.08	4.1E-04
	[66]	8	20 (Cancer)	1.56	7.0E-03
	[83]	5	40 (Squamous)	1.37	1.2E-02
**Colorectal**	[84]	10	5 (Adeno carcinoma)	1.84	8.1E-06
			5 (Cancer)	2.59	1.8E-05
**Prostate**	[85]	17	24 (Cancer)	1.72	6.0E-03
			12 (Prostatic Intraepithelial)	1.69	6.0E-03

**Table:** B. Ly6E is significantly increased in lung, head and neck, ovarian, kidney, melanoma md embryonic tumors than their normal counterparts

Type of cancer	Reference	N (Normal)	N (Cancer)	Fold change	P-value
**Lung**	[29]	20	226 (Adeno)	2.31	8.6E-18
	[86]	28	34 (Squamous)	1.86	2.0E-09
	[87]	10	86 (Adeno)	1.40	4.0E-03
	[28]	30	27 (Adeno)	1.89	4.0E-03
	[88]	25	25 (Adeno)	1.55	8.6E-05
	[27]	58	58 (Adeno)	1.60	2.3E-05
	[26]	49	58 (Adeno)	1.24	9.0E-03
**Head and neck**	[89]	4(Squamous)	16 (Squamous)	3.43	3.1E-07
	[90]	4 (thyroid)	26 (Thyroid papillary)	1.74	6.6E-09
			10 (Tall ceil papillary)	1.67	3.0E-05
			4 (Thyroid anaplastic)	2.22	1.0E-03
	[91]	12 (Tongue)	26 (Tongue squamous)	1.27	5.8E-04
	[92]	22 (Oral cavity)	57 (Squamous)	2.22	2.0E-19
	[92]	10 (Oral cavity)	112 (Squamous)	1.12	1.1E-24
	[23]	9 (Thyroid)	9 (Thyroid papillary)	2.56	5.3E-05
	[93]	4 (Uvula)	34 (Squamous)	1.64	5.2E-04
	[22]	26 (Tongue)	31 (Tongue squamous)	3.67	1.9E-08
	[94]	4 (Thyroid)	14 (Thyroid papillary)	2.72	1.0E-03
	[95]	13 (Buccal mucosa)	41 (Squamous)	2.70	7.1E-07
	[24]	6 (Salivary)	16 (Salivary adenoid)	17.46	3.3E-02
**Ovarian**	[96]	10	43 (Serous adeno)	3.15	5.5E-08
	[97]	4	6 (Serous adeno)	2.31	7.0E-03
	TCGA	8	586 (Serous cyst adeno)	2.81	9.1E-05
	[98]	4	28 (Serous papillary)	1.25	1.6E-02
	[99]	10	85 (Cancer)	1.73	1.8E-04
	[34]	4	41 (Serous adeno)	1.27	1.2E-02
**Kidney**	[43]	5	26 (Clear cell)	4.08	1.4E-07
			4 (Wilms)	2.70	7.0E-03
	[100]	11	32 (Hereditary Clear cell)	4.37	1.1E-10
	[42]	23	8 (Urothelial Carcinoma)	3.00	2.9E-04
			23 (Clear cell)	2.26	2.5E-04
			11 (Papillary)	1.39	2.8E-02
	[101]	3	18 (Wilms)	2.39	5.0E-03
			14 (Clear cell Sarcoma)	1.68	3.3E-02
	[102]	10	10 (clear cell)	1.64	4.9E-02
	[103]	9	9 (Clear cell)	1.63	1.0E-03
**Melanoma**	[104]	7	45 (Cutaneous)	3.02	2.5E-06
**Mixed**	[105]	6 (Normal testis)	9 (Yolk Sac tumor)	5.32	2.7E-08
			15 (Embryonal)	4.74	1.2E-10
	[106]	9	40 (Pleural Malignant Mesothelioma)	4.45	7.8E-06

Data observed using Oncomine (Invitrogen) and G-DOC. N=number of patient samples.

These results show that Ly6E expression was significantly increased in bladder, breast, esophageal, gastric, pancreatic, cervical, colorectal, prostate, lung, head and neck, ovarian, kidney, melanoma, embryonic cancer than their counterpart normal tissues.

The data in Table 4 shows that Ly6E mRNA expression was significantly increased in subtypes of multiple cancers. Ly6E mRNA expression was significantly higher in superficial bladder cancer (n=28) than infiltrating bladder cancer (n=81) and high expression of Ly6E was correlated with higher grade (infiltrating grade 3, n=75 vs infiltrating grade 2, n=6) in Sanchez-Carbayo [17] study. Ly6E mRNA expression was significantly higher in medulloblastoma with CTNNB1 mutation (n=8) than CTNNB1 wildtype tumors (n=38), medulloblastoma with CTNNB1 positive by immunohistochemistry (IHC) (n=6) than CTNNB1 IHC negative (n=44), tumors with MycN amplification (n=14) than tumors not amplified for MycN (n=32) in Kool [107], Robinson [108] and Janoueix-Lerosey [109] studies. Ly6E mRNA expression was significantly higher in esophageal cancer (n=83) than precursor (n=23) in Kimchi [68] and Su [78] studies. Ly6E mRNA expression was significantly higher in pancreatic cancer (n=10) than precursor (n=5) in Logsdon [80] study. High expression of Ly6E was correlated with higher grade of breast cancer. Ly6E mRNA expression was significantly high in ductal N1+ stage (n= 222) than ductal N0 stage (n=274) in Bittner (unpublished, GSE2109), Julka [110], and Ivshina [61], studies and grade 3 tumor (n=334) than grade 1 (n=334) in Loi [111], Buffa [112], Miller [113] and Sotiriou [114] studies, grade 3 (n=64) than grade 2 (n=34) in Bonnefoi [62] study, invasive ductal (n=31) than non-invasive ductal (n=3) in Radvanyi [73] study. Ly6E mRNA expression was significantly higher in tumors with TP53 mutaions (n=130) than tumors with wildtype tumor (n=261) and in tumors with BRCA1 mutaions (n=31) than tumors with BRCA1 wildtype (n=128) in Ivshina [61], Gluck [50], Pawitan [115] studies. (Ly6E) mRNA was found significantly increased in TNBC (n=286) than non-TNBC (n=1653) in Curtis [60], TCGA (Unpublished, NCI), Stickeler [47] and Korde [54] studies.

**Table 4:** Ly6E mRNA expression in subset of multiple cancers

Type of cancer	Reference	Cancer		Fold change	P-value
		N (Group 1)	N (Group 2)		
**Bladder**	[17]	6 (Infiltrating Grade2)	75 (Infiltrating Grade 3)	3.23	3.3E-02
		81 (Infiltrating)	28 (Superficial)	1.50	9.0E-03
	[45]	19 (Alive at 3 years)	33 (dead at 3 years)	1.72	3.9E-02
**Brain and CNS**	[108]	44 (Medulloblastoma CTNNB1 IHC neg)	6 (Medulloblastoma CTNNB1 IHC neg)	2.27	2.0E-03
	[107]	38 (Medulloblastoma CTNNB1 WT)	8 (Medulloblastoma CTNNB1 mutation)	3.11	5.9E-05
	[109]	32(No MycN amplification)	14 (MycN amplification)	1.93	1.1E-02
	[116]	56 (Neuroblastoma No recurrence 5 year)	46 (Neuroblastoma Recurrence 5 year)	1.63	9.4E-06
**Esophageal**	[68]	8 (Precursor)	8 (Cancer)	2.22	3.6E-02
	[78]	15 (Precursor)	75 (Cancer)	1.78	5.0E-03
**Pancreatic**	[80]	5 (Precursor)	10 (Cancer)	3.05	9.4E-06
**Breast**	GSE2109	94 (Ductal N0)	123 (Ductal N1+)	1.46	4.0E-03
	[110]	21 (Ductal N0)	18 (Ductal N1+)	1.46	1.7E-02
	[61]	159 (Ductal N0)	81 (Ductal N1+)	1.27	7.4E-04
	[111]	147(Grade 1)	136(Grade 3)	1.38	1.1E-02
	[112]	42(Grade 1)	65(Grade 3)	1.39	2.0E-02
	[111]	14(Grade 1)	24(Grade 3)	1.53	3.1E-02
	[113]	67(Grade 1)	54(Grade 3)	1.51	2.1E-05
	[114]	64(Grade 1)	55(Grade 3)	1.40	2.0E-03
	[62]	34 (Grade 2)	64 (Grade 3)	1.29	3.0E-02
	[73]	3 (Ductal)	31(Invasive Ductal)	2.25	1.3E-02
	[61]	189 (TP53 wildtype)	58 (TP53 Mutation)	1.30	7.7E-04
	[50]	72 (TP53 wildtype)	72 (TP53 Mutation)	1.25	1.0E-02
	[115]	128 (BRCA1 wildtype)	31 (BRCA1 Mutation)	1.24	1.5E-02
	[60]	1340 (non-TNBC)	211 (TNBC)	1.52	2.9E-21
	TCGA	250 (non-TNBC)	46 (TNBC)	1.55	2.0E-05
	[47]	24 (non-TNBC)	8 (TNBC)	5.28	5.0E-03
	[54]	39 (non-TNBC)	21 (TNBC)	1.51	1.1E-02
	[71]	148 (Metastasis free 1 year)	49 (Metastasis at 1 year)	1.34	2.4E-02
	[117]	171 (Metastasis free 3 year)	19 (Metastasis at 3 year)	1.63	4.0E-03
	[52]	32 (Metastasis free 3 year)	50 (Metastasis at 3 year)	1.64	3.0E-03
	[63]	223 (Metastasis free 3 year)	99 (Metastasis at 3 year)	1.25	4.0E-03
**Gastric**	[118]	7 (Metastasis free 5 years)	11 (Metastasis at 5 year)	2.32	1.4E-02
	[119]	34 (Alive at 1 year)	12 (Dead at 1 year)	1.25	5.0E-03

Ly6E mRNA expression was significantly increased in subtypes of bladder, brain and CNS, esophageal pancreatic, breast and gastric cancer. High Ly6E expression was significantly correlated with poor clinical outcome in bladder, brain and CNS, breast and gastric cancer. Data observed using Oncomine (Invitrogen) and G-DOC. N=number of patient samples.

These results show that Ly6E expression was significantly increased in subtypes of bladder, brain, esophageal, pancreatic and breast cancer.

### High Ly6E expression and survival outcome in multiple cancers

Table 4 also shows a high Ly6E mRNA expression in bladder cancer was significantly correlated with decreased three-year survival (dead, n=33 vs alive, n=19) in Lee [45] study.

High Ly6E mRNA expression in neuroblastoma was significantly correlated with five-year recurrence free survival (recurrence, n=46 vs no recurrence, n=56) in Asgharzadeh [116] study. High Ly6E mRNA expression in breast cancer was significantly correlated with decreased one-year metastasis free survival (metastasis, n=49 vs metastasis free, n=148), decreased three year metastasis free survival (metastasis, n=168 vs metastasis free, n=426) in Bos [71], Schmidt [117], Kao [52] and Hatzis [63] studies. High Ly6E mRNA expression in gastric cancer was significantly correlated with decreased five-year metastasis free survival (metastasis, n=11 vs metastasis free, n=7), decreased one-year overall survival (dead, n=12 vs alive, n=34) in Forster [118] and Chen [119] studies.

**Figure 2:**
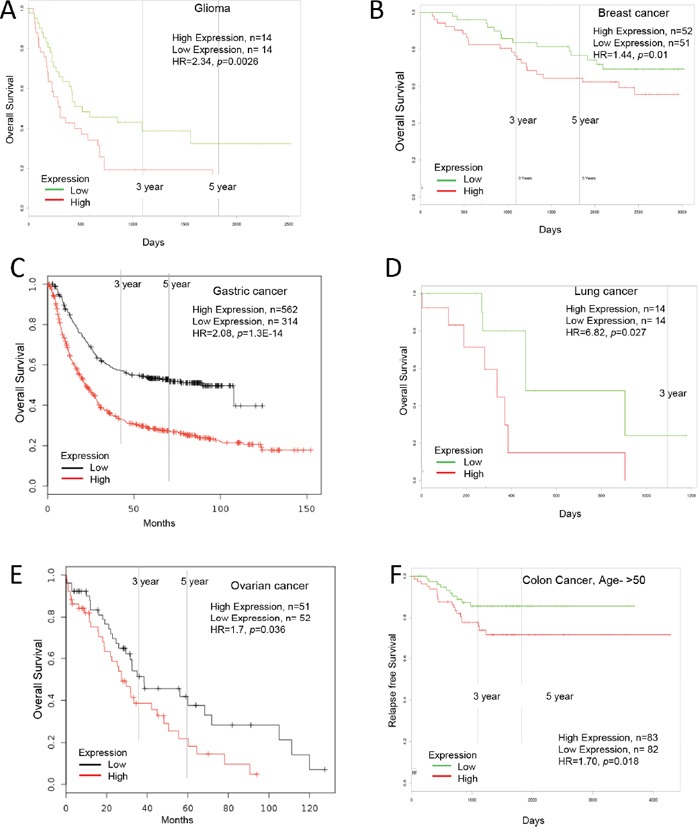
Increased Ly6E expression in cancer and patient survival High Ly6E expression leads to poor survival in **A.** glioma, **B.** breast cancer, **C.** gastric cancer, **D.** lung cancer, **E.** ovarian cancer and **F.** colorectal cancer.

High Ly6E mRNA expression in glioma was significantly correlated with poor five-year overall survival (low Ly6E, n=14; high Ly6E, n=14; HR=2.34, *p*=0.0026, n= number of patient, HR=hazard ratio), shown by PROGgeneV2 (Table S2, Figure 2A). High Ly6E mRNA expression in breast cancer was significantly correlated with poor five-year overall survival with restriction of grade 1 breast cancer (low Ly6E, n=75; high Ly6E, n=60; HR=3.48, *p*=0.022) or without restriction (low Ly6E, n=646; high Ly6E, n=653; HR=1.4, *p*=0.007), five-year relapse free survival with restriction of grade 1 breast cancer (low Ly6E, n=179; high Ly6E, n=129; HR=2.52, *p*=0.001), grade 2 breast cancer (low Ly6E, n=388; high Ly6E, n=336; HR=1.53, *p*=0.001), or without restriction (low Ly6E, n=1113; high Ly6E, n=2441; HR=1.32, *p*=1.50E-05), five-year distant metastasis free survival (low Ly6E, n=479; high Ly6E, n=1130; HR=1.71, *p*=1.90E-05), five-year post progression free survival (low Ly6E, n=99; high Ly6E, n=252; HR=1.43, *p*=0.018), shown by KM plotter. High Ly6E mRNA expression in breast cancer was significantly correlated with poor five-year overall survival with restriction of estrogen receptor positive breast cancer (low Ly6E, n=112; high Ly6E, n=113; HR=1.39, *p*=0.002), with restriction of progesterone receptor positive breast cancer (low Ly6E, n=112; high Ly6E, n=113; HR=1.39, *p*=0.002) or without restriction (low Ly6E, n=15; high Ly6E, n=16; HR=2.73, *p*=0.02), and five-year distance metastasis free survival (low Ly6E, n=112; high Ly6E, n=113; HR=1.44, *p*=0.000) shown by PROGgeneV2 (Table S2, Figure 2B).

High Ly6E mRNA expression in gastric was significantly correlated with poor five-year overall survival (low Ly6E, n=314; high Ly6E, n=562; HR=2.08, *p*=1.3E-14) shown by KM plotter (Table S2, Figure 2C).

High Ly6E mRNA expression in lung cancer was significantly correlated with poor five-year overall survival with restriction of stage IIIa cancer (low Ly6E, n=14; high Ly6E, n=14; HR=6.82, *p*=0.0273) and poor five-year relapse free survival with restriction of stage IIb cancer (low Ly6E, n=22; high Ly6E, n=23; HR=1.97, *p*=0.029) or with restriction of stage 1b cancer (low Ly6E, n=27; high Ly6E, n=27; HR=1.7, *p*=0.046) shown by PROGgeneV2 (Table S2, Figure 2D).

High Ly6E mRNA expression in ovarian cancer was significantly correlated with poor five-year overall survival with restriction of Stage4, serous, grade 3 cancer (low Ly6E, n=52; high Ly6E, n=51; HR=1.7, *p*=0.036) shown by KM plotter (Table S2, Figure 2E).

High Ly6E mRNA expression in colorectal cancer was significantly correlated with poor five-year relapse free survival with restriction of age greater than 50 years (low Ly6E, n=82; high Ly6E, n=83; HR=1.70, *p*=0.018) or without any restriction (low Ly6E, n=93; high Ly6E, n=94; HR=1.77, *p*=0.0005) shown by PROGgeneV2 (Table S2, Figure 2F).

These data show that high Ly6E expression was significantly correlated with poor clinical outcome in glioma, breast, gastric, lung, ovarian and colorectal cancer.

### Increased expression of Ly6H in multiple cancers

To examine the status of Ly6H in human cancer, we used Oncomine or G-DOC to analyze gene expression omnibus (GEO) datasets. As shown in Table 5 we found a significant increased expression of Ly6H in brain and CNS cancer (n=27) than normal relevant tissue (n=10) in Shai [120] and Lee [121] studies. Ly6H mRNA expression was significantly increased in esophageal cancer (n=104) than normal tissue (n=42) in Hao [122] and Kim [123] studies. Ly6H mRNA expression was significantly increased in breast cancer (n=2567) than normal tissue (n=209) in TCGA (unpublished, NCI), Curtis study [20] and Gluck [50] studies. Ly6H mRNA expression was significantly increased in Kidney cancer (n=22) than normal tissue (n=8) in Yusenko [43] and Cutcliffe [101] studies. Ly6H mRNA expression was increased significantly in head and neck cancer (n=20) than normal tissue (n=9) in Schlingemann [124] and Frierson [24] studies. Ly6H mRNA expression was significantly increased in lung cancer (n=47) than normal tissues (n=47) in Bhattacharjee [30] and Su [28] studies. Ly6H mRNA expression was significantly increased in ovarian cancer (n=291) than normal tissues (n=19) in Bonome [35] Lu [125] and Hendrix [34] studies.

**Table 5:** Ly6H mRNA expression in normal and tumor tissue of multiple cancer types

Type of cancer	Reference	N (Normal)	N (Cancer)	Fold change	P-value
**Brain and CNS**	[120]	7 (White matter)	5 (Astrocytoma)	2.66	7.0E-03
	[121]	3 (Neural stem)	22 (Glioblastoma)	1.93	3.8E-02
**Esophageal**	[122]	14	14 (Barrett's)	1.73	9.9E-04
	[123]	28	15 (Barrett's)	1.38	6.5E-06
			75 (Adeno)	1.29	4.3E-08
**Breast**	TCGA	61	36 (Invasive Lobular)	2.72	3.6E-15
			389 (Invasive Ductal)	2.10	6.4E-24
			3 (Male)	3.80	2.5E-02
			4 (Mucinous)	6.18	2.2E-02
			3 (Invasive Ductal and Lobular)	3.10	4.6E-02
			7 (Ductal and Lobular)	2.35	3.0E-03
	[20]	144	10 (Ductal)	1.24	1.2E-02
			46 (Mucinous)	1.61	1.4E-09
			32 (Medullary)	1.20	2.0E-04
			1556 (Invasive Ductal)	1.35	1.0E-55
			148 (Invasive Lobular)	1.37	2.3E-19
			90 (Invasive Ductal and Lobular)	1.34	7.9E-18
			3 (Benign)	1.08	3.0E-02
			14 (Cancer)	1.18	4.6E-04
			5 (Phyllodes)	2.08	1.6E-02
			67 (Tubular)	1.36	7.0E-13
	[50]	4	154 (Invasive)	2.80	5.0E-03
**Kidney**	[43]	5	4 (Wilms)	3.26	1.7E-02
	[101]	3	18 (Wilm)	1.37	1.0E-03
**Head and Neck**	[124]	3 (Hypopharynx)	4 (Squamous)	1.23	3.9E-02
	[24]	6 (Salivary)	16 (Adenoid cystic)	9.13	2.8E-02
**Lung**	[30]	17	20 (Carcinoid)	14.05	3.2E-07
	[28]	30	27 (Adeno)	1.37	2.2E-02
**Ovarian**	[35]	10	185 (Cancer)	1.20	4.0E-07
	[125]	5	7 (Clear cell adeno)	1.19	1.9E-02
	[34]	4	13 (Mucinous adeno)	1.23	1.2E-02
			8 (Clear cell adeno)	1.17	3.1E-02
			37 (Endometrioid adeno)	1.18	2.6E-02
			41 (Serous adeno)	1.15	4.4E-02

Ly6H is significantly increased in brain and CNS, esophageal, breast, kidney, head and neck, lung and ovarian cancer than their normal counterparts. Data observed using Oncomine (Invitrogen). N=number of patient samples.

These results show that Ly6H expression was significantly increased in brain and CNS, esophageal, breast, kidney, head and neck, lung and ovarian cancer than their counterpart normal tissues.

As shown in Table 6, we found that Ly6H mRNA expression was significantly increased in subtypes of multiple cancers. Ly6H mRNA expression was significantly higher in Myc amplified brain and CNS cancer (n= 50) than cancer without Myc amplification (n= 102) in Wang [79] and Robinson [108] studies. Ly6H mRNA expression was significantly higher in estrogen receptor (ER) positive breast cancer (n=562) than ER negative breast cancer (n=244) in Bittner (Unpublished, GSE2109), Wang [126], Stickeler [47] and TCGA (Unpublished, NCI) studies. Ly6H mRNA expression was significantly higher in progesterone receptor (PgR) positive breast cancer (n=215) than PgR negative breast cancer (n=149) in Stickeler [47] and TCGA (Unpublished, NCI) and Chang [127] studies. Ly6H mRNA expression was significantly higher in lobular breast cancer (n=22) than ductal breast cancer (n=188) in Radvanyi [73] and Desmedt [128] studies. Increased Ly6H mRNA expression was significantly correlated with more aggressive phenotype of breast cancer - grade 2 (n=40) than grade 1 (n=40), gastric cancer - stage 3 (n=14) than stage 1 (n=4) in Curtis [129], and Cho [25] studies. Ly6H mRNA expression was significantly increased in clear cell carcinoma of kidney (n=256) than other types (n=59) in TCGA [67] and Bittner(Unpublished, GSE2109) studies. Ly6H mRNA expression was significantly increased in cancerous tissue of cervix (n=8) than precursors cervix cancer (n=23) in Zhai [130] study. Ly6H mRNA expression was significantly increased in colorectal cancer with KRAS mutation (n=27) than KRAS wildtype tumors (n=43) in Khambata-Ford [131] study.

**Table 6:** Ly6H mRNA expression in subset of multiple cancers

Type of cancer	Reference	Cancer		Fold change	P-value
		N (Group 1)	N (Group 2)		
**Brain and CNS**	[79]	81 (Myc not amplified)	20 (Myc amplified)	2.64	9.1E-04
	[108]	21 (Myc not amplified)	30 (Myc amplified)	1.52	1.0E-02
**Breast**	GSE2109	66 (ER neg)	110 (ER pos)	1.24	2.3E-02
	[126]	77 (ER neg)	209 (ER pos)	1.27	2.0E-03
	[47]	14 (ER neg)	18 (ER pos)	2.19	9.0E-03
		15 (PgR neg)	17 (PgR pos)	2.17	1.0E-02
	TCGA	87 (ER neg)	225 (ER pos)	1.31	4.1E-04
		127 (PgR neg)	189 (PgR pos)	1.27	3.7E-04
	[127]	7 (PgR neg)	9 (PgR pos)	2.41	4.6E-02
	[129]	40 (Grade 1)	26 (Grade 2)	1.21	6.0E-03
	[73]	30 (Ductal)	5 (Lobular)	2.35	1.0E-03
	[128]	158 (Ductal)	17 (Lobular)	1.28	2.6E-02
		105 (Recurrence free at 5 year)	47 (Recurrence at 5 year)	1.25	1.0E-02
		123 (Metastasis free at 5 year)	29 (Metastasis at 5 year)	1.23	1.0E-02
**Gastric**	[25]	4 (Stage 1)	14 (Stage 3)	1.56	3.0E-03
**Kidney**	GSE2109	43 (Others)	184 (Clear cell)	1.43	4.0E-03
	TCGA	16 (Papillary)	72 (Clear cell)	1.68	4.3E-05
**Pancreatic**	[140]	23 (Precursor)	8 (Cancer)	1.22	2.0E-02
**Cervical**	[130]	7 (Precursor)	21 (Cancer)	1.37	5.0E-03
**Colorectal**	[131]	43 (KRAS wildtype)	27 (KRAS Mutation)	1.64	4.0E-03
	[65]	30 (Recurrence free at 5 year)	56 (Recurrence at 5 year)	1.24	3.3E-02

Ly6H mRNA expression was significantly increased in subtypes of brain and CNS, breast, gastric, kidney, pancreatic, cervical and colorectal cancer. High Ly6H expression was significantly correlated with poor clinical outcome in breast and colorectal cancer. Data observed using Oncomine (Invitrogen). N=number of patient samples.

These results show that Ly6H expression was significantly increased in subtypes of brain and CNS, breast, gastric, kidney, pancreatic, cervical, and colorectal cancer.

### High Ly6H expression and survival outcome in multiple cancers

Table 6 also showed that a high Ly6H mRNA expression in breast cancer was significantly correlated with decreased five-year recurrence survival (recurrence, n=47 vs recurrence free, n=105) and decreased five-year metastasis free survival (metastasis, n=29 vs metastasis free, n=123) in Desmedt [128] study. High Ly6H mRNA expression in colorectal cancer was significantly correlated with decreased five-year recurrence survival (recurrence, n=56 vs recurrence free, n=30) in Jorissen [65] study.

**Figure 3:**
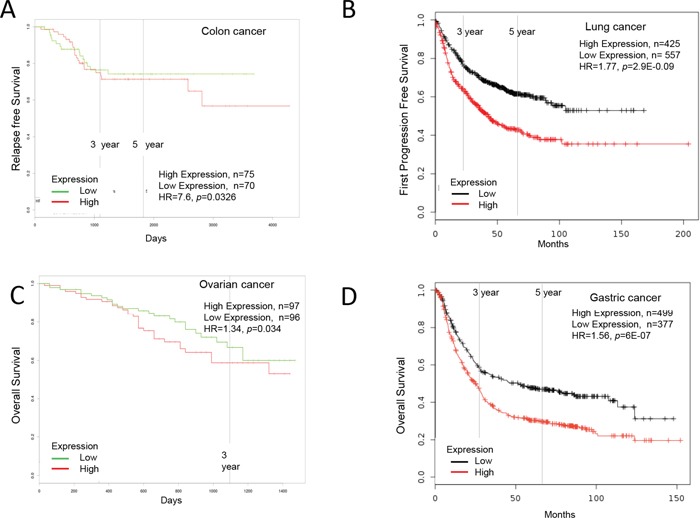
Increased Ly6H expression in cancer and patient survival High Ly6H expression leads to poor survival in **A.** colon cancer, **B.** lung cancer, **C.** ovarian cancer and **D.** gastric cancer.

High Ly6H mRNA expression in colorectal cancer was significantly correlated with poor five-year relapse free survival (low Ly6H, n=70; high Ly6E, n=70; HR=7.6, *p*=0.0326, n= number of patient, HR=hazard ratio) shown by PROGgeneV2 (Table S3, Figure 3A).

High Ly6H mRNA expression in lung cancer was significantly correlated with poor five-year first progression free survival (low Ly6H, n=557; high Ly6E, n=425; HR=1.77, *p*=2.90E-09), five-year post progression free survival (low Ly6H, n=87; high Ly6E, n=257; HR=1.47, *p*=0.015) and five-year overall survival (low Ly6H, n=484; high Ly6E, n=1442; HR=1.3, *p*=6.00E-04) shown by KM plotter (Table S3, Figure 3B).

High Ly6H mRNA expression in ovarian cancer was significantly correlated with poor five-year post progression free survival (low Ly6H, n=305; high Ly6E, n=506; HR=1.3, *p*=6.00E-04) shown by KM plotter and five-year overall survival (low Ly6H, n=96; high Ly6E, n=97; HR=1.34, *p*=0.034) shown by PROGgeneV2 (Table S3, Figure 3C).

High Ly6H mRNA expression in gastric cancer was significantly correlated with poor five-year first progression free survival (low Ly6H, n=336; high Ly6E, n=499; HR=1.5, *p*=6.9E-05) and five-year overall survival (low Ly6H, n=377; high Ly6E, n=499; HR=1.56, *p*=6E-07) shown by KM plotter (Table S3, Figure 3D).

These data show that high Ly6H expression was significantly correlated with poor clinical outcome in in breast, colon, lung, ovarian and gastric cancer.

### Increased expression of Ly6K in multiple cancers

We investigated whether Ly6K was differentially expressed in clinical samples of cancer in multiple studies. The data was visualized using Oncomine. As shown in Table 7, we found that Ly6K mRNA expression was significantly increased in 188 samples of bladder cancer than 68 samples of normal relevant tissue in Lee [45] studies. Ly6K mRNA expression was significantly increased in 497 samples of breast cancer than 205 samples of normal tissue in TCGA (Unpublished, NCI) and Curtis [60] study. Ly6K mRNA expression was significantly increased in 40 samples of cervical cancer than 5 samples of normal tissue in Biewenga [83] study. Ly6K mRNA expression was significantly increased in 51 samples of esophageal cancer than 51 samples of normal tissue in Su [78] study. Ly6K mRNA expression was significantly increased in 92 samples of head and neck cancer than 43 samples of normal tissue in Peng [92], He [23] and Ye [91] studies. Ly6K mRNA expression was significantly increased in 375 samples of lung cancer than 143 samples of normal tissue in Hou [31], Selamat [27], Okayama [29] studies. Ly6K mRNA expression was significantly increased in 273 samples of colorectal cancer than 71 samples of normal tissue in Sabates-Bellver [38], TCGA [132] and Skrzypczak [41] studies.

**Table 7:** Ly6K mRNA expression in normal and tumor tissue of multiple cancer types

Type of cancer	Reference	N (Normal)	N (Cancer)	Fold change	P-value
**Bladder**	[45]	68	62 (Infiltrating)	1.30	8.0E-07
			126 (Superficial)	1.20	5.1E-08
**Breast**	TCGA	61	76 (Invasive)	1.20	2.4E-08
			389 (Invasive Ductal)	1.32	3.2E-08
	[60]	144	32 (medullary)	1.08	3.0E-02
**Cervical**	[83]	5	40 (Squamous)	4.97	3.2E-11
**Esphageal**	[78]	51	51 (Squamous)	1.65	1.8E-06
**Head and neck**	[92]	22 (Oral cavity)	57 (Squamous)	1.79	1.4E-08
	[23]	9 (Thyroid)	9 (Papillary)	1.15	3.0E-03
	[91]	12 (Tongue)	26 (Squamous)	1.16	6.0E-03
**Lung**	[31]	65	27 (Squamous)	6.06	6.8E-12
			19 (Large cell)	1.09	1.2E-04
			45 (Adeno)	3.18	4.0E-07
	[27]	58	58 (Adeno)	1.04	7.0E-03
	[29]	20	226 (Adeno)	1.38	8.7E-04
**Colorectal**	[38]	7	7 (Rectal adeno)	1.82	3.0E-03
	TCGA [132]	19	29 (Colon Muc adeno)	1.21	9.0E-03
		3	24 (Cecum adeno)	1.27	2.0E-03
		3	6 (Rectal Muc adeno)	1.38	2.0E-03
		3	60 (Rectal adeno)	1.34	9.2E-04
		19	102 (Colon adeno)	1.29	9.1E-05
	[41]	24	45 (Colon adeno)	1.21	5.0E-13

Ly6K is significantly increased in bladder, breast, cervical, esophageal, head and neck, lung and colorectal cancer than their normal counterparts. Data observed using Oncomine (Invitrogen). N=number of patient samples.

These results show that Ly6K expression was significantly increased in bladder, breast, cervical, esophageal, head and neck, lung and colorectal cancer than their counter part normal tissue.

As shown in Table 8, we found that Ly6K expression was increased in subtypes of multiple cancers. Ly6K mRNA expression was significantly higher in Myc amplified brain and CNS cancer (n=12) than cancer without Myc amplification (n=5) in Robinson [108] study. Ly6K mRNA expression was significantly higher in astrocytoma, grade 4 (n=76) than astrocytoma grade 3 (n=24) in Phillips [69] study. Ly6K expression was significantly higher in glioblastoma (n=59) than astrocytoma (n=8) in Freije [133] study. Ly6K expression was significantly higher in recurred brain tumors (n=7) than primary brain tumors (n=20) in Liang [134] study. Higher Ly6K expression was correlated with breast cancer stage as seen by the significant higher expression of Ly6K in ductal stage N1+ (n=19) than ductal stage N0 (n=20) and invasive stage N1+ (n=9) than invasive stage N0 n=22) in Julka [110], and Stickeler [47] studies. Ly6K mRNA expression was significantly higher in triple negative breast cancer (TNBC) (n=163) than non-TNBC breast cancer (n=584) in Bittner (Unpublished, GSE2109), Korde [54], TCGA (Unpublished, NCI), Julka [110], Zhao [135], Richardson2 [55] and Miyake [136] studies. Ly6K mRNA expression was significantly higher in gastric cancer grade 3 (n=18) than grade 2 (n=6) in Forster [118] study. Ly6K mRNA expression was significantly higher in TP53 mutated lung cancer (n=18) than TP53 wildtye cancer (n=23), grade 3 adenocarcinoma (n=14) than grade 2 adenocarcinoma (n=18) in Ding [137] study. Ly6K mRNA expression was significantly higher in cohort of squamous lung carcinoma (n=309) than cohorts of non small cell lung carcinoma (n=218) in TCGA [138], Bild [51], Lee [139], and Hou [31] studies. High Ly6K expression was significantly correlated with higher cancer staging in squamous lung cancer stage N1+ (n=12) than stage N0 (n=25) and in colorectal adenocarcinoma (n=15) than N0 stage in Bittner(Unpublished, GSE2109), study. Ly6K mRNA expression was significantly higher in cohort of ovarian cancer (n=8) than cohorts of precursors (n=24) in Buchholz [140] study and ovarian cancer (n=361) than borderline tumor (n=57) in Bittner(Unpublished, GSE2109), Anglesio [141] and Tothill [142] studies.

**Table 8:** Ly6K mRNA is significantly increased in subset of multiple cancer subtypes

Type of cancer	Reference	Cancer		Fold change	P-value
		N (Group 1)	N (Group 2)		
**Bladder**	[45]	11 (Alive at 5 years)	33 (Dead at 5 years)	1.20	3.9E-02
**Brain and CNS**	[108]	5 (No Myc amplification)	12 (Myc amplification)	1.26	2.5E-02
	[69]	24 (Astrocytoma Grade 3)	76 (Astrocytoma Grade 4)	1.38	2.2E-02
	[134]	20 (Primary)	7 (Recurred)	1.35	1.4E-02
	[133]	8 (Astrocytoma)	59 Glioblastoma	1.22	5.0E-03
		3 (Glioma alive at 3 year)	6 (Dead at 3 year)	1.49	9.0E-03
**Kidney**	[143]	55 (Alive at 1 year)	16 (Dead at 1 year)	2.11	1.2E-02
	TCGA [67]	9 (Alive at 5 year)	12 (Dead at 5 year)	1.77	3.1E-02
**Breast**	[110]	20 (Ductal stage N0)	19 (Ductal stageN1+)	1.64	3.0E-03
	[47]	22 (Invasive stage N0)	9 (Invasive stage N1+)	4.02	6.0E-03
	GSE2109	129 (Non-TNBC)	39 (TNBC)	2.97	1.1E-05
	[54]	39 (Non-TNBC)	21 (TNBC)	2.45	1.0E-03
	TCGA	250 (Non-TNBC)	46 (TNBC)	2.82	9.2E-05
	[52]	30 (Non-TNBC)	8 (TNBC)	2.01	1.0E-02
	[135]	28 (Non-TNBC)	5 (TNBC)	2.38	3.7E-02
	[55]	19 (Non-TNBC)	18 (TNBC)	3.74	1.0E-02
	[136]	89 (Non-TNBC)	26 (TNBC)	2.32	1.7E-02
	[135]	295 (Alive at 3 year)	31 (Dead at 3 year)	1.47	2.3E-02
	[115]	156 (Alive at 1 year)	3 (Dead at 1 year)	1.63	3.3E-02
**Gastric**	[118]	6 (Grade 2)	18 (Grade 3)	4.52	1.4E-02
**Lung**	[137]	18 (Adeno, grade 2)	14 (Adeno, grade 3)	5.48	1.2E-04
		23 (TP53 wildtype)	18 (TP53 mutation)	2.23	3.3E-02
	TCGA [138]	33 (Non-small cell)	154 (Squamous)	2.74	2.0E-03
	[51]	58 (Non-small cell)	53 (Squamous)	2.23	2.0E-03
	[139]	63 (Non-small cell)	75 (Squamous cell)	2.17	6.0E-03
	[31]	64 (Non-small cell)	27 (Squamous cell)	2.39	1.5E-04
	GSE2109	25 (Squamous N0)	12 (Squamous N1+)	2.02	1.9E-02
**Colorectal**	GSE2109	17 (Colon adeno N0)	15 (Colon adeno N1+)	2.00	4.5E-02
**Pancreas**	[140]	24 (Precursor)	8 (Cancer)	1.68	2.0E-03
**Ovarian**	GSE2109	10 (Borderline tumor)	146 (Cancer)	1.64	5.2E-04
	[141]	30 (Borderline tumor)	44 (Cancer)	1.54	5.0E-03
	[142]	17 (Borderline tumor)	171 (Cancer)	1.70	4.1E-06
		5 (5-year recurrence free)	103 (Recurrence at 5-year)	1.60	4.1E-02

Ly6K mRNA expression was significantly increased in subtypes of bladder, brain and CNS, kidney, breast, gastric, lung, colorectal, pancreatic and ovarian cancer. High Ly6K expression was significantly correlated with poor clinical outcome in bladder, brain and CNS, Kidney, breast, and ovarian cancer. Data observed using Oncomine (Invitrogen). N=number of patient samples.

These results show that Ly6K expression was significantly increased in subtypes of brain and CNS, breast, gastric, lung, colorectal, pancreatic, and ovarian cancer.

### High Ly6K expression and survival outcome in multiple cancers

Table 8 also showed a high Ly6K mRNA expression in bladder cancer was significantly correlated with decreased five-year overall survival (dead, n=33 vs alive, n=11) in Lee [45] study. High Ly6K mRNA expression in brain and CNS cancer was significantly correlated with decreased three-year overall survival (dead, n=33 vs alive, n=11) in Freije [133] study. High Ly6K mRNA expression in kidney cancer was significantly correlated with decreased one-year overall survival (dead, n=16 vs alive, n=55) and five-year overall survival (dead, n=12 vs alive, n=9) in Zhao [143] and TCGA [67] studies respectively. High Ly6K mRNA expression in breast cancer was significantly correlated with decreased three-year overall survival (dead, n=31 vs alive, n=295) and one-year overall survival (dead, n=3 vs alive n=156) in Kao [52] and Pawitan [115] studies.

**Figure 4:**
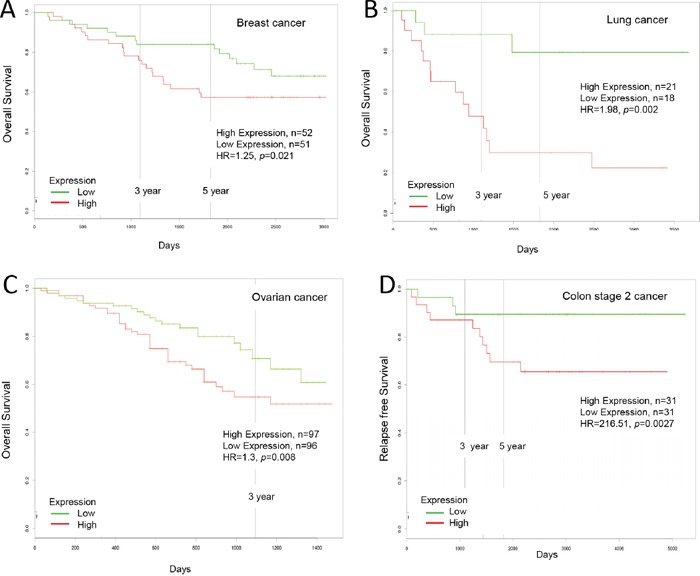
Increased Ly6K expression in cancer and patient survival High Ly6H expression leads to poor survival in **A.** breast cancer, **B.** lung cancer, **C.** ovarian cancer and **D.** colon cancer.

High Ly6K mRNA expression in breast cancer was significantly correlated with poor five-year overall survival (low Ly6K, n=51; high Ly6K, n=52; HR=1.25, *p*=0.021, HR=hazard ratio, n=number of patients) shown by PROGgeneV2 (Table S4, Figure 4A). High Ly6K mRNA expression in lung cancer was significantly correlated with poor five-year relapse free survival with restriction of stage IIb cancer (low Ly6K, n=18; high Ly6K, n=21; HR=2.02, *p*=0.002); five year overall survival with restriction of stage IIIb cancer (low Ly6K, n=17; high Ly6K, n=18; HR=1.57, *p*=0.013) and with restriction of stage IIb cancer (low Ly6K, n=18; high Ly6K, n=21; HR=1.98, *p*=0.002) shown by PROGgeneV2 (Table S4, Figure 4B). High Ly6K mRNA expression in ovarian cancer was significantly correlated with poor five-year overall survival (low Ly6K, n=96; high Ly6K, n=97; HR=1.3, *p*=0.0008) shown by PROGgeneV2 (Table S4, Figure 4C). High Ly6K mRNA expression in colorectal cancer was highly correlated but not significantly associated with poor five-year relapse free survival (low Ly6K, n=60; high Ly6K, n=61; HR=13.81, *p*=0.059) shown by PROGgeneV2 (Table S4, Figure 4D).

These data show that high Ly6K expression was significantly correlated with poor clinical outcome in bladder, brain and CNS, kidney, breast, lung and ovarian cancer.

## DISCUSSION

In this study we show that Ly6 family members (Ly6D, Ly6E, Ly6H, Ly6K) are up regulated in cancerous tissue than normal tissue, the Increased expression of these genes are heterogeneous among difference subtypes of multiple cancer and that the high expression of these genes is significantly associated with poor outcome. Recently Butte et al described that the gene expression data of tumor mass can be influenced by infiltration by immune cells and non-cancerous normal cells [144]. These contaminations may affect the analysis for gene signature associated with tumor, specifically for cells which are comprise a very little percentage of total tumor mass such as tumor infiltrating immune cells. In this study we focused on comparison of tumor vs normal. So the normal cell contamination in tumor may downplay the increased expression of Ly6 genes in tumor tissue than normal. However we observed a consistent increased expression of Ly6 in multiple studies of pan cancer. The mRNA expression data across multiple studies shows that ovarian, colorectal, gastric, breast, lung, brain and CNS, cervical, esophageal, head and neck and pancreatic cancers express significant high levels of Ly6D, Ly6E, Ly6H, Ly6K. As summarized in Table 9, the gene expression analysis showed that bladder cancer expresses significant high levels of Ly6D, Ly6E, Ly6K. The survival data for colorectal, ovarian and gastric cancer showed that all four studied genes Ly6D, Ly6E, Ly6H, Ly6K are poor prognosis markers for multiple cancer types. Survival data for bladder and brain and CNS cancer showed that Ly6E and Ly6K is poor prognosis marker for these cancers. Survival data for breast and lung cancer show that Ly6D, Ly6E and Ly6K were poor prognosis marker for these cancers. Survival data for cervical, esophageal, head and neck and pancreatic cancers in public databases were either non significant or were not available.

**Table 9:** Correlation of high mRNA expression and patient survival outcome in multiple cancer types

Cancer Type	Genes	Expression in Tumors (*p*<0.05)	Survival Analysis (*p*<0.05)
**Ovarian**	LY6D	up	Poor prognosis
	LY6E	up	Poor prognosis
	LY6H	up	Poor prognosis
	LY6K	up	Poor prognosis
**Colorectal**	LY6D	up	Poor prognosis
	LY6E	up	Poor prognosis
	LY6H	up	Poor prognosis
	LY6K	up	Poor prognosis
**Gastric**	LY6D	up	Poor prognosis
	LY6E	up	Poor prognosis
	LY6H	up	Poor prognosis
	LY6K	up	Poor prognosis
**Breast**	LY6D	up	Poor prognosis
	LY6E	up	Poor prognosis
	LY6H	up	Poor Prognosis
	LY6K	up	Poor prognosis
**Lung**	LY6D	up	Poor prognosis
	LY6E	up	Poor prognosis
	LY6H	up	OS (NS), Others (NA)
	LY6K	up	Poor prognosis
**Bladder**	LY6D	up	OS (NS), Others (NA)
	LY6E	up	Poor prognosis
	LY6H	NS	OS (NS), Others (NA)
	LY6K	up	Poor prognosis
**Brain and CNS**	LY6D	up	OS (NS), Others (NA)
	LY6E	up	Poor prognosis
	LY6H	up	OS (NS), Others (NA)
	LY6K	up	Poor prognosis
**Cervical**	LY6D	up	OS (NA), RFS (NS)
	LY6E	up	OS (NA), RFS (NS)
	LY6H	up	OS (NA), RFS (NS)
	LY6K	up	OS (NA), RFS (NS)
**Esophageal**	LY6D	up	OS (NS), Others (NA)
	LY6E	up	OS (NS), Others (NA)
	LY6H	up	OS (NS), Others (NA)
	LY6K	up	OS (NS), Others (NA)
**Head and neck**	LY6D	up	OS (NS), Others (NA)
	LY6E	up	OS (NS), Others (NA)
	LY6H	up	OS (NS), Others (NA)
	LY6K	up	OS (NS), Others (NA)
**Pancreatic**	LY6D	up	OS (NS), Others (NA)
	LY6E	up	OS (NS), Others (NA)
	LY6H	up	OS (NS), Others (NA)
	LY6K	up	OS (NS), Others (NA)

OS overall survival, RFS relapse free survival, NS not significant *p*<0.05, NA no data available.

**Figure 5:**
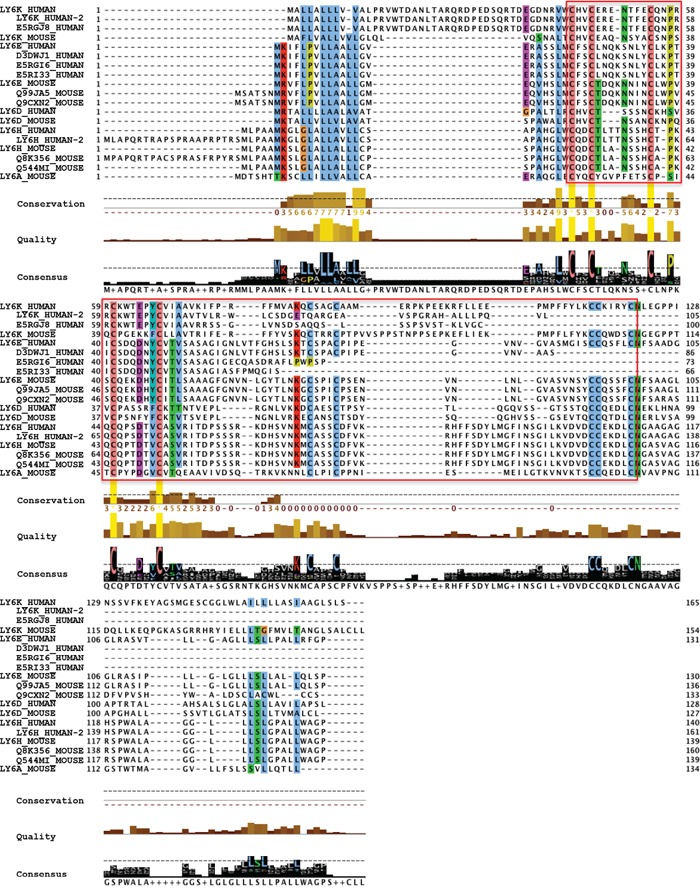
Ly6 gene family members have conserved LU/uPAR domain The red box shows the region containing multiple cysteine residues that form di-sulfide bonds characteristic of the LU/uPAR domain in Ly6 family of proteins. Highlighting shows columns were the consensus sequence is present in over 50% of the aligned amino acids.

**Figure 6:**
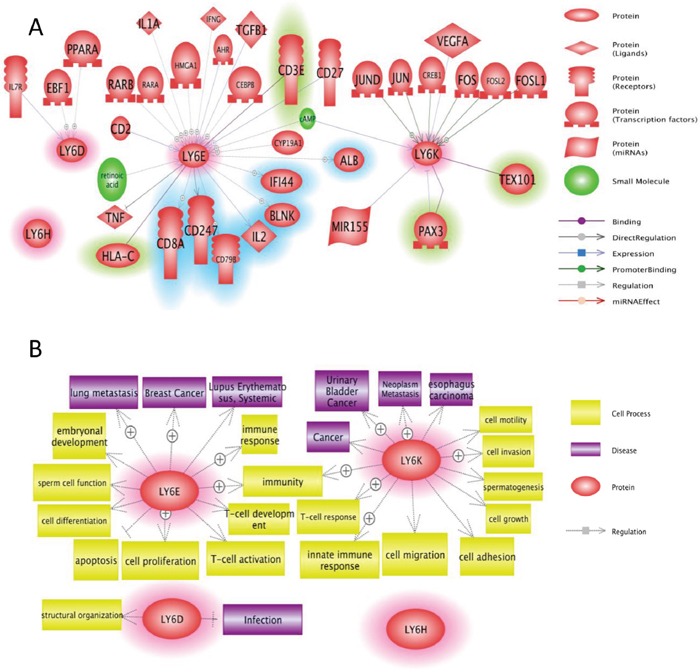
Network analysis of Ly6 gene family members **A.** Pathway studio network analysis showed that Ly6 signaling is involved in broad range of molecules including growth factor, nuclear receptor, and micro RNAs. The upstream regulators are not highlighted, the downstream effectors are highlighted with blue, and the potential binding partners are highlighted with green. **B.** Pathway studio network analysis showed that Ly6 gene family affect multitude of cellular fate and cell-cell interaction with microenvironment ranging from growth, apoptosis, autophagy, immune response.

The mouse and human Ly6 family have a conserved LU domain (Figure 5). The LU domain is described as three-fold repeated domain in urokinase-type plasminogen activated receptors (uPAR), which occurs singly in Ly6 family [145-147]. The uPAR signaling is responsible for initiating invasion and metastasis via the activation of the plasminogen activator/plasmin cascade in breast cancers and play a role in stimulating the RAS/ERK pathway to control invasion in cancer cells [148]. The LU domain has been predicted to play a role in cancer diagnosis and malfunction of immune system [149]. It is plausible that all Ly6 family of genes may have common mechanism in tumorigenesis and their role in poor prognosis. The four genes are clustered closely at Chr8q24, the predicted transcription factor binding to their respective proximal promoter within 10KB of start site may be driven by common regulatory elements. Recently experimental validation of the important role for AP-1 activation in promoting LY6K gene expression was observed, whereas the SNP242 C allele or methylation of the CpG site was associated with reduced Ly6K expression via inhibition of AP1 [150], suggesting additional level of complexities in regulation of Ly6 genes. Further more Ly6K, Ly6E and Ly6D expression may be regulated by multiple growth factors, nuclear receptors (Figure 6A) that can affect multitude of cellular fate including immune response, cell motility, growth, adhesion and differentiation (Figure 6B).

The Ly6K and Ly6E proteins are implicated as cancer vaccine targets and drug conjugated antibody therapy, respectively [7-9] suggesting that Ly6 family members can be used as novel candidates to develop targeted therapies. However, a detailed understanding of mechanism associated with Ly6 function is lacking. We had previously shown that Sca-1/Ly6A in mouse tumor model can inhibit TGF-β signaling by direct binding with TGF-β receptor 1 [2]. We have tested for human Ly6E and Ly6K in breast cancer and delineated the molecular mechanism behind cancer cell growth, metastasis and drug resistance (being published separately). This may explain how various members of Ly6 gene family may be responsible for poor outcome in multiple cancers. The protein level validation for all four proteins in pan cancer is yet to be determined. We are currently in process of generating antibodies and validating commercially available antibodies using control and knockout cell lines. The protein expression of the all four genes will need to be done in pan cancer clinical samples in future studies, so that Ly6 gene family can be used as a companion diagnostic tool for multiple cancer types. The Ly6 family of genes can be a novel prognosis marker and novel candidate to develop targeted therapies.

## MATERIALS AND METHODS

### Bioinformatic analysis

ONCOMINE (www.oncomine.org) [151, 152] was used to visualize mRNA expression of Ly6 gene family members in different cancer types. CBioPortal (www.cbioportal.org) [153, 154] for Cancer Genomics was used to explore genetic alterations across Ly6 gene family members in different cancer types. When selecting genomic profiles, mutations and CNAs are specified by default. When available, survival analysis of LY6K and its isoforms genetic alteration in specific cancer types were selected. PathwayStudio (http://www.elsevier.com/solutions/pathway-studio) [155] was used to generate LY6K and its isoforms signaling pathway. Km plotter (kmplot.com/analysis) [156] was used to collect information about survival analysis of LY6K and its isoforms in pan-cancers. Oncomine (Invitrogen), KM plotter (http://kmplot.com/analysis/) [156] and ProgeneV2 prognostic Database (http://www.abren.net/PrognoScan/) [157] were used to collect information about survival analysis of LY6K and its isoforms in pan-cancers.

### Alignment of Ly6 proteins

Protein sequences of the LY6K, LY6E, LY6D, LY6H genes from both human and mouse plus the Ly6A gene from mouse were obtained from UniProtKB [158]. In addition to the predominant protein forms, selected isoforms for each protein were also obtained. Sequences were aligned using Clustal Omega [159] with default parameters. The alignment was performed using editing and analysis into Jalview [160] platform. In the alignment presentation the small fragment proteins, redundant sequences and truncated isoforms were excluded.

## SUPPLEMENTARY FIGURES AND TABLES





## References

[1] Klonisch T, Wiechec E, Hombach-Klonisch S, Ande SR, Wesselborg S, Schulze-Osthoff K, Los M (2008). Cancer stem cell markers in common cancers - therapeutic implications. Trends Mol Med.

[2] Upadhyay G, Yin Y, Yuan H, Li X, Derynck R, Glazer RI (2010). Stem cell antigen-1 enhances tumorigenicity by disruption of growth differentiation factor-10 (GDF10)-dependent TGF-{beta} signaling. Proc Natl Acad Sci USA.

[3] McKenzie IF, Gardiner J, Cherry M, Snell GD (1977). Lymphocyte antigens: Ly-4, Ly-6, and Ly-7. Transplant Proc.

[4] Woody JN, Feldmann M, Beverley PC, McKenzie IF (1977). Expression of alloantigens LY-5 and LY-6 on cytotoxic effector cells. J Immunol.

[5] Lee PY, Wang J-X, Parisini E, Dascher CC, Nigrovic PA (2013). Ly6 family proteins in neutrophil biology. J Leukoc Biol.

[6] Matsuda R, Enokida H, Chiyomaru T, Kikkawa N, Sugimoto T, Kawakami K, Tatarano S, Yoshino H, Toki K, Uchida Y, Kawahara K, Nishiyama K, Seki N (2011). LY6K is a novel molecular target in bladder cancer on basis of integrate genome-wide profiling. Brit J Cancer.

[7] Asundi J, Crocker L, Tremayne J, Chang P, Sakanaka C, Tanguay J, Spencer S, Chalasani S, Luis E, Gascoigne K, Desai R, Raja R, Friedman BA (2015). An Antibody-Drug Conjugate Directed against Lymphocyte Antigen 6 Complex, Locus E (LY6E) Provides Robust Tumor Killing in a Wide Range of Solid Tumor Malignancies. Clin Cancer Res.

[8] Ishikawa H, Imano M, Shiraishi O, Yasuda A, Peng Y-F, Shinkai M, Yasuda T, Imamoto H, Shiozaki H (2014). Phase I clinical trial of vaccination with LY6K-derived peptide in patients with advanced gastric cancer. Gastric Cancer.

[9] Kong HK, Park JH (2012). Characterization and function of human Ly-6/uPAR molecules. BMB Rep.

[10] Mallya M, Campbell RD, Aguado B (2002). Transcriptional analysis of a novel cluster of LY-6 family members in the human and mouse major histocompatibility complex: five genes with many splice forms. Genomics.

[11] Davies A, Simmons DL, Hale G, Harrison RA, Tighe H, Lachmann PJ, Waldmann H (1989). CD59, an LY-6-like protein expressed in human lymphoid cells, regulates the action of the complement membrane attack complex on homologous cells. J Exp Med.

[12] Naylor TL, Greshock J, Wang Y, Colligon T, Yu QC, Clemmer V, Zaks TZ, Weber BL (2005). High resolution genomic analysis of sporadic breast cancer using array-based comparative genomic hybridization. Breast Cancer Res.

[13] Grisanzio C, Freedman ML (2010). Chromosome 8q24-Associated Cancers and MYC. Genes Cancer.

[14] Asundi J, Crocker L, Tremayne J, Chang P, Sakanaka C, Tanguay J, Spencer S, Chalasani S, Luis E, Gascoigne K, Desai R, Raja R, Friedman BA (2015). An Antibody-Drug Conjugate Directed against Lymphocyte Antigen 6 Complex, Locus E (LY6E) Provides Robust Tumor Killing in a Wide Range of Solid Tumor Malignancies. Clin Cancer Res.

[15] Yoshitake Y, Fukuma D, Yuno A, Hirayama M, Nakayama H, Tanaka T, Nagata M, Takamune Y, Kawahara K, Nakagawa Y, Yoshida R, Hirosue A, Ogi H (2015). Phase II Clinical Trial of Multiple Peptide Vaccination for Advanced Head and Neck Cancer Patients Revealed Induction of Immune Responses and Improved OS. Clin Cancer Res.

[16] Cheng C-W, Hsiao J-R, Fan C-C, Lo Y-K, Tzen C-Y, Wu L-W, Fang W-Y, Cheng A-J, Chen C-H, Chang I-S, Jiang SS, Chang J-Y, Lee AY-L (2015). Loss of GDF10/BMP3b as a prognostic marker collaborates with TGFBR3 to enhance chemotherapy resistance and epithelial-mesenchymal transition in oral squamous cell carcinoma. Mol Carcinog.

[17] Sanchez-Carbayo M, Socci ND, Lozano J, Saint F, Cordon-Cardo C (2006). Defining molecular profiles of poor outcome in patients with invasive bladder cancer using oligonucleotide microarrays. Journal of clinical oncology.

[18] Dyrskjot L, Kruhoffer M, Thykjaer T, Marcussen N, Jensen JL, Moller K, Orntoft TF (2004). Gene expression in the urinary bladder: a common carcinoma in situ gene expression signature exists disregarding histopathological classification. Cancer Res.

[19] Sun L, Hui AM, Su Q, Vortmeyer A, Kotliarov Y, Pastorino S, Passaniti A, Menon J, Walling J, Bailey R, Rosenblum M, Mikkelsen T, Fine HA (2006). Neuronal and glioma-derived stem cell factor induces angiogenesis within the brain. Cancer Cell.

[20] Curtis C, Shah SP, Chin S-F, Turashvili G, Rueda OM, Dunning MJ, Speed D, Lynch AG, Samarajiwa S, Yuan Y, Gräf S, Ha G, Haffari G (2012). The genomic and transcriptomic architecture of 2,000 breast tumours reveals novel subgroups. Nature.

[21] Lin CY, Vega VB, Thomsen JS, Zhang T, Kong SL, Xie M, Chiu KP, Lipovich L, Barnett DH, Stossi F, Yeo A, George J, Kuznetsov VA (2007). Whole-genome cartography of estrogen receptor alpha binding sites. PLoS Genet.

[22] Estilo CL, P OC, Talbot S, Socci ND, Carlson DL, Ghossein R, Williams T, Yonekawa Y, Ramanathan Y, Boyle JO, Kraus DH, Patel S, Shaha AR (2009). Oral tongue cancer gene expression profiling: Identification of novel potential prognosticators by oligonucleotide microarray analysis. BMC Cancer.

[23] He H, Jazdzewski K, Li W, Liyanarachchi S, Nagy R, Volinia S, Calin GA, Liu CG, Franssila K, Suster S, Kloos RT, Croce CM, la Chapelle de A (2005). The role of microRNA genes in papillary thyroid carcinoma. Proc Natl Acad Sci USA.

[24] Frierson HFJ, El-Naggar AK, Welsh JB, Sapinoso LM, Su AI, Cheng J, Saku T, Moskaluk CA, Hampton GM (2002). Large scale molecular analysis identifies genes with altered expression in salivary adenoid cystic carcinoma. The American journal of pathology.

[25] Cho JY, Lim JY, Cheong JH, Park YY, Yoon SL, Kim SM, Kim SB, Kim H, Hong SW, Park YN, Noh SH, Park ES, Chu IS (2011). Gene expression signature-based prognostic risk score in gastric cancer. Clin Cancer Res.

[26] Landi MT, Dracheva T, Rotunno M, Figueroa JD, Liu H, Dasgupta A, Mann FE, Fukuoka J, Hames M, Bergen AW, Murphy SE, Yang P, Pesatori AC (2008). Gene expression signature of cigarette smoking and its role in lung adenocarcinoma development and survival. Plos One.

[27] Selamat SA, Chung BS, Girard L, Zhang W, Zhang Y, Campan M, Siegmund KD, Koss MN, Hagen JA, Lam WL, Lam S, Gazdar AF, Laird-Offringa IA (2012). Genome-scale analysis of DNA methylation in lung adenocarcinoma and integration with mRNA expression. Genome research.

[28] Su LJ, Chang CW, Wu YC, Chen KC, Lin CJ, Liang SC, Lin CH, Whang-Peng J, Hsu SL, Chen CH, Huang CY (2007). Selection of DDX5 as a novel internal control for Q-RT-PCR from microarray data using a block bootstrap re-sampling scheme. BMC genomics.

[29] Okayama H, Kohno T, Ishii Y, Shimada Y, Shiraishi K, Iwakawa R, Furuta K, Tsuta K, Shibata T, Yamamoto S, Watanabe S, Sakamoto H, Kumamoto K (2012). Identification of genes upregulated in ALK-positive and EGFR/KRAS/ALK-negative lung adenocarcinomas. Cancer Res.

[30] Bhattacharjee A, Richards WG, Staunton J, Li C, Monti S, Vasa P, Ladd C, Beheshti J, Bueno R, Gillette M, Loda M, Weber G, Mark EJ (2001). Classification of human lung carcinomas by mRNA expression profiling reveals distinct adenocarcinoma subclasses. Proc Natl Acad Sci USA.

[31] Hou J, Aerts J, Hamer den B, van Ijcken W, Bakker den M, Riegman P, van der Leest C, van der Spek P, Foekens JA, Hoogsteden HC, Grosveld F, Philipsen S (2010). Gene expression-based classification of non-small cell lung carcinomas and survival prediction. Plos One.

[32] Wachi S, Yoneda K, Wu R (2005). Interactome-transcriptome analysis reveals the high centrality of genes differentially expressed in lung cancer tissues. Bioinformatics.

[33] Welsh JB, Zarrinkar PP, Sapinoso LM, Kern SG, Behling CA, Monk BJ, Lockhart DJ, Burger RA, Hampton GM (2001). Analysis of gene expression profiles in normal and neoplastic ovarian tissue samples identifies candidate molecular markers of epithelial ovarian cancer. Proc Natl Acad Sci USA.

[34] Hendrix ND, Wu R, Kuick R, Schwartz DR, Fearon ER, Cho KR (2006). Fibroblast growth factor 9 has oncogenic activity and is a downstream target of Wnt signaling in ovarian endometrioid adenocarcinomas. Cancer Res.

[35] Bonome T, Levine DA, Shih J, Randonovich M, Pise-Masison CA, Bogomolniy F, Ozbun L, Brady J, Barrett JC, Boyd J, Birrer MJ (2008). A gene signature predicting for survival in suboptimally debulked patients with ovarian cancer. Cancer Res.

[36] Pei H, Li L, Fridley BL, Jenkins GD, Kalari KR, Lingle W, Petersen G, Lou Z, Wang L (2009). FKBP51 affects cancer cell response to chemotherapy by negatively regulating Akt. Cancer Cell.

[37] Badea L, Herlea V, Dima SO, Dumitrascu T, Popescu I (2008). Combined gene expression analysis of whole-tissue and microdissected pancreatic ductal adenocarcinoma identifies genes specifically overexpressed in tumor epithelia. Hepato-gastroenterology.

[38] Sabates-Bellver J, Van der Flier LG, de Palo M, Cattaneo E, Maake C, Rehrauer H, Laczko E, Kurowski MA, Bujnicki JM, Menigatti M, Luz J, Ranalli TV, Gomes V (2007). Transcriptome profile of human colorectal adenomas. Molecular cancer research.

[39] Kaiser S, Park YK, Franklin JL, Halberg RB, Yu M, Jessen WJ, Freudenberg J, Chen X, Haigis K, Jegga AG, Kong S, Sakthivel B, Xu H (2007). Transcriptional recapitulation and subversion of embryonic colon development by mouse colon tumor models and human colon cancer. Genome Biol.

[40] Gaedcke J, Grade M, Jung K, Camps J, Jo P, Emons G, Gehoff A, Sax U, Schirmer M, Becker H, Beissbarth T, Ried T, Ghadimi BM (2010). Mutated KRAS results in overexpression of DUSP4, a MAP-kinase phosphatase, and SMYD3, a histone methyltransferase, in rectal carcinomas. Genes, chromosomes & cancer.

[41] Skrzypczak M, Goryca K, Rubel T, Paziewska A, Mikula M, Jarosz D, Pachlewski J, Oledzki J, Ostrowski J (2010). Modeling oncogenic signaling in colon tumors by multidirectional analyses of microarray data directed for maximization of analytical reliability. Plos One.

[42] Jones J, Otu H, Spentzos D, Kolia S, Inan M, Beecken WD, Fellbaum C, Gu X, Joseph M, Pantuck AJ, Jonas D, Libermann TA (2005). Gene signatures of progression and metastasis in renal cell cancer. Clin Cancer Res.

[43] Yusenko MV, Kuiper RP, Boethe T, Ljungberg B, van Kessel AG, Kovacs G (2009). High-resolution DNA copy number and gene expression analyses distinguish chromophobe renal cell carcinomas and renal oncocytomas. BMC Cancer.

[44] Stransky N, Vallot C, Reyal F, Bernard-Pierrot I, de Medina SG, Segraves R, de Rycke Y, Elvin P, Cassidy A, Spraggon C, Graham A, Southgate J, Asselain B (2006). Regional copy number-independent deregulation of transcription in cancer. Nat Genet.

[45] Lee JS, Leem SH, Lee SY, Kim SC, Park ES, Kim SB, Kim SK, Kim YJ, Kim WJ, Chu IS (2010). Expression signature of E2F1 and its associated genes predict superficial to invasive progression of bladder tumors. Journal of clinical oncology.

[46] Pomeroy SL, Tamayo P, Gaasenbeek M, Sturla LM, Angelo M, McLaughlin ME, Kim JY, Goumnerova LC, Black PM, Lau C, Allen JC, Zagzag D, Olson JM (2002). Prediction of central nervous system embryonal tumour outcome based on gene expression. Nature.

[47] Stickeler E, Pils D, Klar M, Orlowsk-Volk M, Hausen Zur A, Jager M, Watermann D, Gitsch G, Zeillinger R, Tempfer CB (2011). Basal-like molecular subtype and HER4 up-regulation and response to neoadjuvant chemotherapy in breast cancer. Oncol Rep.

[48] Minn AJ, Gupta GP, Siegel PM, Bos PD, Shu W, Giri DD, Viale A, Olshen AB, Gerald WL, Massague J (2005). Genes that mediate breast cancer metastasis to lung. Nature.

[49] Waddell N, Cocciardi S, Johnson J, Healey S, Marsh A, Riley J, da Silva L, Vargas AC, Reid L, kConFab I, Simpson PT, Lakhani SR, Chenevix-Trench G (2010). Gene expression profiling of formalin-fixed, paraffin-embedded familial breast tumours using the whole genome-DASL assay. J Pathol.

[50] Gluck S, Ross JS, Royce M, McKenna EFJ, Perou CM, Avisar E, Wu L (2012). TP53 genomics predict higher clinical and pathologic tumor response in operable early-stage breast cancer treated with docetaxel-capecitabine +/− trastuzumab. Breast Cancer Res Treat.

[51] Bild AH, Yao G, Chang JT, Wang Q, Potti A, Chasse D, Joshi MB, Harpole D, Lancaster JM, Berchuck A, Olson JAJ, Marks JR, Dressman HK (2006). Oncogenic pathway signatures in human cancers as a guide to targeted therapies. Nature.

[52] Kao KJ, Chang KM, Hsu HC, Huang AT (2011). Correlation of microarray-based breast cancer molecular subtypes and clinical outcomes: implications for treatment optimization. BMC Cancer.

[53] Farmer P, Bonnefoi H, Becette V, Tubiana-Hulin M, Fumoleau P, Larsimont D, Macgrogan G, Bergh J, Cameron D, Goldstein D, Duss S, Nicoulaz AL, Brisken C (2005). Identification of molecular apocrine breast tumours by microarray analysis. Oncogene.

[54] Korde LA, Lusa L, McShane L, Lebowitz PF, Lukes L, Camphausen K, Parker JS, Swain SM, Hunter K, Zujewski JA (2010). Gene expression pathway analysis to predict response to neoadjuvant docetaxel and capecitabine for breast cancer. Breast Cancer Res Treat.

[55] Richardson AL, Wang ZC, De Nicolo A, Lu X, Brown M, Miron A, Liao X, Iglehart JD, Livingston DM, Ganesan S (2006). X chromosomal abnormalities in basal-like human breast cancer. Cancer Cell.

[56] Esserman LJ, Berry DA, Cheang MC, Yau C, Perou CM, Carey L, DeMichele A, Gray JW, Conway-Dorsey K, Lenburg ME, Buxton MB, Davis SE, van't Veer LJ (2012). Chemotherapy response and recurrence-free survival in neoadjuvant breast cancer depends on biomarker profiles: results from the I-SPY 1 TRIAL (CALGB 150007/150012; ACRIN 6657). Breast Cancer Res Treat.

[57] Chin K, DeVries S, Fridlyand J, Spellman PT, Roydasgupta R, Kuo WL, Lapuk A, Neve RM, Qian Z, Ryder T, Chen F, Feiler H, Tokuyasu T (2006). Genomic and transcriptional aberrations linked to breast cancer pathophysiologies. Cancer Cell.

[58] Ginestier C, Cervera N, Finetti P, Esteyries S, Esterni B, Adelaide J, Xerri L, Viens P, Jacquemier J, Charafe-Jauffret E, Chaffanet M, Birnbaum D, Bertucci F (2006). Prognosis and gene expression profiling of 20q13-amplified breast cancers. Clin Cancer Res.

[59] van't Veer LJ, Dai HY, van de Vijver MJ, He Y, Hart A, Mao M, Peterse HL, van der Kooy K, Marton MJ, Witteveen AT, Schreiber GJ, Kerkhoven RM, Roberts C (2002). Gene expression profiling predicts clinical outcome of breast cancer. Nature.

[60] Curtis C, Shah SP, Chin SF, Turashvili G, Rueda OM, Dunning MJ, Speed D, Lynch AG, Samarajiwa S, Yuan Y, Graf S, Ha G, Haffari G (2012). The genomic and transcriptomic architecture of 2,000 breast tumours reveals novel subgroups. Nature.

[61] Ivshina AV, George J, Senko O, Mow B, Putti TC, Smeds J, Lindahl T, Pawitan Y, Hall P, Nordgren H, Wong JE, Liu ET, Bergh J (2006). Genetic reclassification of histologic grade delineates new clinical subtypes of breast cancer. Cancer Res.

[62] Bonnefoi H, Potti A, Delorenzi M, Mauriac L, Campone M, Tubiana-Hulin M, Petit T, Rouanet P, Jassem J, Blot E, Becette V, Farmer P, Andre S (2007). Validation of gene signatures that predict the response of breast cancer to neoadjuvant chemotherapy: a substudy of the EORTC 10994/BIG 00-01 clinical trial. The Lancet Oncology.

[63] Hatzis C, Pusztai L, Valero V, Booser DJ, Esserman L, Lluch A, Vidaurre T, Holmes F, Souchon E, Wang H, Martin M, Cotrina J, Gomez H (2011). A genomic predictor of response and survival following taxane-anthracycline chemotherapy for invasive breast cancer. JAMA.

[64] D'Errico M, de Rinaldis E, Blasi MF, Viti V, Falchetti M, Calcagnile A, Sera F, Saieva C, Ottini L, Palli D, Palombo F, Giuliani A, Dogliotti E (2009). Genome-wide expression profile of sporadic gastric cancers with microsatellite instability. European journal of cancer.

[65] Jorissen RN, Lipton L, Gibbs P, Chapman M, Desai J, Jones IT, Yeatman TJ, East P, Tomlinson IP, Verspaget HW, Aaltonen LA, Kruhoffer M, Orntoft TF (2008). DNA copy-number alterations underlie gene expression differences between microsatellite stable and unstable colorectal cancers. Clin Cancer Res.

[66] Pyeon D, Newton MA, Lambert PF, Boon den JA, Sengupta S, Marsit CJ, Woodworth CD, Connor JP, Haugen TH, Smith EM, Kelsey KT, Turek L. P., Ahlquist P. (2007). Fundamental differences in cell cycle deregulation in human papillomavirus-positive and human papillomavirus-negative head/neck and cervical cancers. Cancer Res.

[67] Cancer Genome Atlas Research N (2013). Comprehensive molecular characterization of clear cell renal cell carcinoma. Nature.

[68] Kimchi ET, Posner MC, Park JO, Darga TE, Kocherginsky M, Karrison T, Hart J, Smith KD, Mezhir JJ, Weichselbaum RR, Khodarev NN (2005). Progression of Barrett's metaplasia to adenocarcinoma is associated with the suppression of the transcriptional programs of epidermal differentiation. Cancer Res.

[69] Phillips HS, Kharbanda S, Chen R, Forrest WF, Soriano RH, Wu TD, Misra A, Nigro JM, Colman H, Soroceanu L, Williams PM, Modrusan Z, Feuerstein BG (2006). Molecular subclasses of high-grade glioma predict prognosis, delineate a pattern of disease progression, and resemble stages in neurogenesis. Cancer Cell.

[70] Collisson EA, Sadanandam A, Olson P, Gibb WJ, Truitt M, Gu S, Cooc J, Weinkle J, Kim GE, Jakkula L, Feiler HS, Ko AH, Olshen AB (2011). Subtypes of pancreatic ductal adenocarcinoma and their differing responses to therapy. Nature medicine.

[71] Bos PD, Zhang XH, Nadal C, Shu W, Gomis RR, Nguyen DX, Minn AJ, van de Vijver MJ, Gerald WL, Foekens JA, Massague J (2009). Genes that mediate breast cancer metastasis to the brain. Nature.

[72] Smith JJ, Deane NG, Wu F, Merchant NB, Zhang B, Jiang A, Lu P, Johnson JC, Schmidt C, Bailey CE, Eschrich S, Kis C, Levy S (2010). Experimentally derived metastasis gene expression profile predicts recurrence and death in patients with colon cancer. Gastroenterology.

[73] Radvanyi L, Singh-Sandhu D, Gallichan S, Lovitt C, Pedyczak A, Mallo G, Gish K, Kwok K, Hanna W, Zubovits J, Armes J, Venter D, Hakimi J (2005). The gene associated with trichorhinophalangeal syndrome in humans is overexpressed in breast cancer. Proc Natl Acad Sci USA.

[74] Ma XJ, Dahiya S, Richardson E, Erlander M, Sgroi DC (2009). Gene expression profiling of the tumor microenvironment during breast cancer progression. Breast Cancer Res.

[75] Gluck S, Ross JS, Royce M, McKenna EFJ, Perou CM, Avisar E, Wu L (2012). TP53 genomics predict higher clinical and pathologic tumor response in operable early-stage breast cancer treated with docetaxel-capecitabine +/− trastuzumab. Breast Cancer Res Treat.

[76] Zhao H, Langerød A, Ji Y, Nowels KW, Nesland JM, Tibshirani R, Bukholm IK, Kåresen R, Botstein D, Børresen-Dale A-L, Jeffrey SS (2004). Different gene expression patterns in invasive lobular and ductal carcinomas of the breast. Mol Biol Cell.

[77] Hu N, Clifford RJ, Yang HH, Wang C, Goldstein AM, Ding T, Taylor PR, Lee MP (2010). Genome wide analysis of DNA copy number neutral loss of heterozygosity (CNNLOH) and its relation to gene expression in esophageal squamous cell carcinoma. BMC genomics.

[78] Su H, Hu N, Yang HH, Wang C, Takikita M, Wang Q-H, Giffen C, Clifford R, Hewitt SM, Shou J-Z, Goldstein AM, Lee MP, Taylor PR (2011). Global Gene Expression Profiling and Validation in Esophageal Squamous Cell Carcinoma and Its Association with Clinical Phenotypes. Clin Cancer Res.

[79] Wang Q, Diskin S, Rappaport E, Attiyeh E, Mosse Y, Shue D, Seiser E, Jagannathan J, Shusterman S, Bansal M, Khazi D, Winter C, Okawa E (2006). Integrative genomics identifies distinct molecular classes of neuroblastoma and shows that multiple genes are targeted by regional alterations in DNA copy number. Cancer Res.

[80] Logsdon CD, Simeone DM, Binkley C, Arumugam T, Greenson JK, Giordano TJ, Misek DE, Kuick R, Hanash S (2003). Molecular profiling of pancreatic adenocarcinoma and chronic pancreatitis identifies multiple genes differentially regulated in pancreatic cancer. Cancer Res.

[81] Pei H, Li L, Fridley BL, Jenkins GD, Kalari KR, Lingle W, Petersen G, Lou Z, Wang L (2009). FKBP51 affects cancer cell response to chemotherapy by negatively regulating Akt. Cancer Cell.

[82] Scotto L, Narayan G, Nandula SV, Arias-Pulido H, Subramaniyam S, Schneider A, Kaufmann AM, Wright JD, Pothuri B, Mansukhani M, Murty VV (2008). Identification of copy number gain and overexpressed genes on chromosome arm 20q by an integrative genomic approach in cervical cancer: potential role in progression. Genes, chromosomes & cancer.

[83] Biewenga P, Buist MR, Moerland PD, Ver Loren van Themaat E, van Kampen AH, Kate ten FJ, Baas F (2008). Gene expression in early stage cervical cancer. Gynecologic oncology.

[84] Skrzypczak M, Goryca K, Rubel T, Paziewska A, Mikula M, Jarosz D, Pachlewski J, Oledzki J, Ostrowski J, Ostrowsk J (2010). Modeling oncogenic signaling in colon tumors by multidirectional analyses of microarray data directed for maximization of analytical reliability. Plos One.

[85] Tomlins SA, Mehra R, Rhodes DR, Cao X, Wang L, Dhanasekaran SM, Kalyana-Sundaram S, Wei JT, Rubin MA, Pienta KJ, Shah RB, Chinnaiyan AM (2007). Integrative molecular concept modeling of prostate cancer progression. Nat Genet.

[86] Talbot SG, Estilo C, Maghami E, Sarkaria IS, Pham DK, P OC, Socci ND, Ngai I, Carlson D, Ghossein R, Viale A, Park BJ, Rusch VW (2005). Gene expression profiling allows distinction between primary and metastatic squamous cell carcinomas in the lung. Cancer Res.

[87] Beer DG, Kardia SL, Huang CC, Giordano TJ, Levin AM, Misek DE, Lin L, Chen G, Gharib TG, Thomas DG, Lizyness ML, Kuick R, Hayasaka S (2002). Gene-expression profiles predict survival of patients with lung adenocarcinoma. Nature medicine.

[88] Wei TY, Juan CC, Hisa JY, Su LJ, Lee YC, Chou HY, Chen JM, Wu YC, Chiu SC, Hsu CP, Liu KL, Yu CT (2012). Protein arginine methyltransferase 5 is a potential oncoprotein that upregulates G1 cyclins/cyclin-dependent kinases and the phosphoinositide 3-kinase/AKT signaling cascade. Cancer Sci.

[89] Toruner GA, Ulger C, Alkan M, Galante AT, Rinaggio J, Wilk R, Tian B, Soteropoulos P, Hameed MR, Schwalb MN, Dermody JJ (2004). Association between gene expression profile and tumor invasion in oral squamous cell carcinoma. Cancer genetics and cytogenetics.

[90] Giordano TJ, Au AY, Kuick R, Thomas DG, Rhodes DR, Wilhelm KGJ, Vinco M, Misek DE, Sanders D, Zhu Z, Ciampi R, Hanash S, Chinnaiyan A (2006). Delineation, functional validation, and bioinformatic evaluation of gene expression in thyroid follicular carcinomas with the PAX8-PPARG translocation. Clin Cancer Res.

[91] Ye H, Yu T, Temam S, Ziober BL, Wang J, Schwartz JL, Mao L, Wong DT, Zhou X (2008). Transcriptomic dissection of tongue squamous cell carcinoma. BMC genomics.

[92] Peng CH, Liao CT, Peng SC, Chen YJ, Cheng AJ, Juang JL, Tsai CY, Chen TC, Chuang YJ, Tang CY, Hsieh WP, Yen TC (2011). A novel molecular signature identified by systems genetics approach predicts prognosis in oral squamous cell carcinoma. Plos One.

[93] Cromer A, Carles A, Millon R, Ganguli G, Chalmel F, Lemaire F, Young J, Dembele D, Thibault C, Muller D, Poch O, Abecassis J, Wasylyk B (2004). Identification of genes associated with tumorigenesis and metastatic potential of hypopharyngeal cancer by microarray analysis. Oncogene.

[94] Vasko V, Espinosa AV, Scouten W, He H, Auer H, Liyanarachchi S, Larin A, Savchenko V, Francis GL, la Chapelle de A, Saji M, Ringel MD (2007). Gene expression and functional evidence of epithelial-to-mesenchymal transition in papillary thyroid carcinoma invasion. Proc Natl Acad Sci USA.

[95] Ginos MA, Page GP, Michalowicz BS, Patel KJ, Volker SE, Pambuccian SE, Ondrey FG, Adams GL, Gaffney PM (2004). Identification of a gene expression signature associated with recurrent disease in squamous cell carcinoma of the head and neck. Cancer Res.

[96] Yoshihara K, Tajima A, Komata D, Yamamoto T, Kodama S, Fujiwara H, Suzuki M, Onishi Y, Hatae M, Sueyoshi K, Fujiwara H, Kudo Y, Inoue I (2009). Gene expression profiling of advanced-stage serous ovarian cancers distinguishes novel subclasses and implicates ZEB2 in tumor progression and prognosis. Cancer Sci.

[97] Adib TR, Henderson S, Perrett C, Hewitt D, Bourmpoulia D, Ledermann J, Boshoff C (2004). Predicting biomarkers for ovarian cancer using gene-expression microarrays. Br J Cancer.

[98] Welsh JB, Zarrinkar PP, Sapinoso LM, Kern SG, Behling CA, Monk BJ, Lockhart DJ, Burger RA, Hampton GM (2001). Analysis of gene expression profiles in normal and neoplastic ovarian tissue samples identifies candidate molecular markers of epithelial ovarian cancer. Proc Natl Acad Sci USA.

[99] Bonome T, Levine DA, Shih J, Randonovich M, Pise-Masison CA, Bogomolniy F, Ozbun L, Brady J, Barrett JC, Boyd J, Birrer MJ (2008). A gene signature predicting for survival in suboptimally debulked patients with ovarian cancer. Cancer Res.

[100] Beroukhim R, Brunet J-P, Di Napoli A, Mertz KD, Seeley A, Pires MM, Linhart D, Worrell RA, Moch H, Rubin MA, Sellers WR, Meyerson M, Linehan WM (2009). Patterns of gene expression and copy-number alterations in von-hippel lindau disease-associated and sporadic clear cell carcinoma of the kidney. Cancer Res.

[101] Cutcliffe C, Kersey D, Huang C-C, Zeng Y, Walterhouse D, Perlman EJ (2005). Clear cell sarcoma of the kidney: up-regulation of neural markers with activation of the sonic hedgehog and Akt pathways. Clin Cancer Res.

[102] Gumz ML, Zou H, Kreinest PA, Childs AC, Belmonte LS, LeGrand SN, Wu KJ, Luxon BA, Sinha M, Parker AS, Sun L-Z, Ahlquist DA, Wood CG (2007). Secreted frizzled-related protein 1 loss contributes to tumor phenotype of clear cell renal cell carcinoma. Clin Cancer Res.

[103] Lenburg ME, Liou LS, Gerry NP, Frampton GM, Cohen HT, Christman MF (2003). Previously unidentified changes in renal cell carcinoma gene expression identified by parametric analysis of microarray data. BMC Cancer.

[104] Talantov D, Mazumder A, Yu JX, Briggs T, Jiang Y, Backus J, Atkins D, Wang Y (2005). Novel genes associated with malignant melanoma but not benign melanocytic lesions. Clin Cancer Res.

[105] Korkola JE, Houldsworth J, Chadalavada RS, Olshen AB, Dobrzynski D, Reuter VE, Bosl GJ, Chaganti RS (2006). Down-regulation of stem cell genes, including those in a 200-kb gene cluster at 12p13. 31, is associated with in vivo differentiation of human male germ cell tumors. Cancer Res.

[106] Gordon GJ, Rockwell GN, Jensen RV, Rheinwald JG, Glickman JN, Aronson JP, Pottorf BJ, Nitz MD, Richards WG, Sugarbaker DJ, Bueno R (2005). Identification of novel candidate oncogenes and tumor suppressors in malignant pleural mesothelioma using large-scale transcriptional profiling. The American journal of pathology.

[107] Kool M, Koster J, Bunt J, Hasselt NE, Lakeman A, van Sluis P, Troost D, Meeteren NS, Caron HN, Cloos J, Mrsic A, Ylstra B, Grajkowska W (2008). Integrated genomics identifies five medulloblastoma subtypes with distinct genetic profiles, pathway signatures and clinicopathological features. Plos One.

[108] Robinson G, Parker M, Kranenburg TA, Lu C, Chen X, Ding L, Phoenix TN, Hedlund E, Wei L, Zhu X, Chalhoub N, Baker SJ, Huether R (2012). Novel mutations target distinct subgroups of medulloblastoma. Nature.

[109] Janoueix-Lerosey I, Lequin D, Brugieres L, Ribeiro A, de Pontual L, Combaret V, Raynal V, Puisieux A, Schleiermacher G, Pierron G, Valteau-Couanet D, Frebourg T, Michon J (2008). Somatic and germline activating mutations of the ALK kinase receptor in neuroblastoma. Nature.

[110] Julka PK, Chacko RT, Nag S, Parshad R, Nair A, Oh DS, Hu Z, Koppiker CB, Nair S, Dawar R, Dhindsa N, Miller ID, Ma D (2008). A phase II study of sequential neoadjuvant gemcitabine plus doxorubicin followed by gemcitabine plus cisplatin in patients with operable breast cancer: prediction of response using molecular profiling. Br J Cancer.

[111] Loi S, Haibe-Kains B, Desmedt C, Wirapati P, Lallemand F, Tutt AM, Gillet C, Ellis P, Ryder K, Reid JF, Daidone MG, Pierotti MA, Berns EM (2008). Predicting prognosis using molecular profiling in estrogen receptor-positive breast cancer treated with tamoxifen. BMC genomics.

[112] Buffa FM, Camps C, Winchester L, Snell CE, Gee HE, Sheldon H, Taylor M, Harris AL, Ragoussis J (2011). microRNA-associated progression pathways and potential therapeutic targets identified by integrated mRNA and microRNA expression profiling in breast cancer. Cancer Res.

[113] Miller LD, Smeds J, George J, Vega VB, Vergara L, Ploner A, Pawitan Y, Hall P, Klaar S, Liu ET, Bergh J (2005). An expression signature for p53 status in human breast cancer predicts mutation status, transcriptional effects, and patient survival. Proc Natl Acad Sci USA.

[114] Sotiriou C, Neo S-Y, McShane LM, Korn EL, Long PM, Jazaeri A, Martiat P, Fox SB, Harris AL, Liu ET (2003). Breast cancer classification and prognosis based on gene expression profiles from a population-based study. Proc Natl Acad Sci USA.

[115] Pawitan Y, Bjohle J, Amler L, Borg AL, Egyhazi S, Hall P, Han X, Holmberg L, Huang F, Klaar S, Liu ET, Miller L, Nordgren H (2005). Gene expression profiling spares early breast cancer patients from adjuvant therapy: derived and validated in two population-based cohorts. Breast Cancer Res.

[116] Asgharzadeh S, Pique-Regi R, Sposto R, Wang H, Yang Y, Shimada H, Matthay K, Buckley J, Ortega A, Seeger RC (2006). Prognostic significance of gene expression profiles of metastatic neuroblastomas lacking MYCN gene amplification. Journal of the National Cancer Institute.

[117] Schmidt AM, Yan SD, Yan SF, Stern DM (2000). The biology of the receptor for advanced glycation end products and its ligands. Biochim Biophys Acta.

[118] Forster S, Gretschel S, Jons T, Yashiro M, Kemmner W (2011). THBS4, a novel stromal molecule of diffuse-type gastric adenocarcinomas, identified by transcriptome-wide expression profiling. Modern pathology.

[119] Chen X, Leung SY, Yuen ST, Chu K-M, Ji J, Li R, Chan ASY, Law S, Troyanskaya OG, Wong J, So S, Botstein D, Brown PO (2003). Variation in gene expression patterns in human gastric cancers. Mol Biol Cell.

[120] Shai R, Shi T, Kremen TJ, Horvath S, Liau LM, Cloughesy TF, Mischel PS, Nelson SF (2003). Gene expression profiling identifies molecular subtypes of gliomas. Oncogene.

[121] Lee J, Kotliarova S, Kotliarov Y, Li A, Su Q, Donin NM, Pastorino S, Purow BW, Christopher N, Zhang W, Park JK, Fine HA (2006). Tumor stem cells derived from glioblastomas cultured in bFGF and EGF more closely mirror the phenotype and genotype of primary tumors than do serum-cultured cell lines. Cancer Cell.

[122] Hao Y, Triadafilopoulos G, Sahbaie P, Young HS, Omary MB, Lowe AW (2006). Gene expression profiling reveals stromal genes expressed in common between Barrett's esophagus and adenocarcinoma. Gastroenterology.

[123] Kim SM, Park Y-Y, Park ES, Cho JY, Izzo JG, Zhang Di, Kim S-B, Lee JH, Bhutani MS, Swisher SG, Wu X, Coombes KR, Maru D (2010). Prognostic biomarkers for esophageal adenocarcinoma identified by analysis of tumor transcriptome. Plos One.

[124] Schlingemann J, Habtemichael N, Ittrich C, Toedt G, Kramer H, Hambek M, Knecht R, Lichter P, Stauber R, Hahn M (2005). Patient-based cross-platform comparison of oligonucleotide microarray expression profiles. Lab Invest.

[125] Lu KH, Patterson AP, Wang L, Marquez RT, Atkinson EN, Baggerly KA, Ramoth LR, Rosen DG, Liu J, Hellstrom I, Smith D, Hartmann L, Fishman D (2004). Selection of potential markers for epithelial ovarian cancer with gene expression arrays and recursive descent partition analysis. Clin Cancer Res.

[126] Wang Y, Klijn JGM, Zhang Y, Sieuwerts AM, Look MP, Yang F, Talantov D, Timmermans M, Gelder MEM-V, Yu J, Jatkoe T, Berns EMJJ, Atkins D (2005). Gene-expression profiles to predict distant metastasis of lymph-node-negative primary breast cancer. Lancet.

[127] Chang JC, Wooten EC, Tsimelzon A, Hilsenbeck SG, Gutierrez MC, Elledge R, Mohsin S, Osborne CK, Chamness GC, Allred DC, O'Connell P (2003). Gene expression profiling for the prediction of therapeutic response to docetaxel in patients with breast cancer. Lancet.

[128] Desmedt C, Piette F, Loi S, Wang Y, Lallemand F, Haibe-Kains B, Viale G, Delorenzi M, Zhang Y, d'Assignies MS, Bergh J, Lidereau R, Ellis P (2007). Strong time dependence of the 76-gene prognostic signature for node-negative breast cancer patients in the TRANSBIG multicenter independent validation series. Clin Cancer Res.

[129] Curtis C, Shah SP, Chin SF, Turashvili G, Rueda OM, Dunning MJ, Speed D, Lynch AG, Samarajiwa S, Yuan Y, Graf S, Ha G, Haffari G (2012). The genomic and transcriptomic architecture of 2,000 breast tumours reveals novel subgroups. Nature.

[130] Zhai Y, Kuick R, Nan B, Ota I, Weiss SJ, Trimble CL, Fearon ER, Cho KR (2007). Gene expression analysis of preinvasive and invasive cervical squamous cell carcinomas identifies HOXC10 as a key mediator of invasion. Cancer Res.

[131] Khambata-Ford S, Garrett CR, Meropol NJ, Basik M, Harbison CT, Wu S, Wong TW, Huang X, Takimoto CH, Godwin AK, Tan BR, Krishnamurthi SS, Burris HA (2007). Expression of epiregulin and amphiregulin and K-ras mutation status predict disease control in metastatic colorectal cancer patients treated with cetuximab. Journal of clinical oncology.

[132] Cancer Genome Atlas N (2012). Comprehensive molecular characterization of human colon and rectal cancer. Nature.

[133] Freije WA, Castro-Vargas FE, Fang Z, Horvath S, Cloughesy T, Liau LM, Mischel PS, Nelson SF (2004). Gene expression profiling of gliomas strongly predicts survival. Cancer Res.

[134] Liang Y, Diehn M, Watson N, Bollen AW, Aldape KD, Nicholas MK, Lamborn KR, Berger MS, Botstein D, Brown PO, Israel MA (2005). Gene expression profiling reveals molecularly and clinically distinct subtypes of glioblastoma multiforme. Proc Natl Acad Sci USA.

[135] Zhao H, Langerod A, Ji Y, Nowels KW, Nesland JM, Tibshirani R, Bukholm IK, Karesen R, Botstein D, Borresen-Dale AL, Jeffrey SS (2004). Different gene expression patterns in invasive lobular and ductal carcinomas of the breast. Mol Biol Cell.

[136] Miyake T, Nakayama T, Naoi Y, Yamamoto N, Otani Y, Kim SJ, Shimazu K, Shimomura A, Maruyama N, Tamaki Y, Noguchi S (2012). GSTP1 expression predicts poor pathological complete response to neoadjuvant chemotherapy in ER-negative breast cancer. Cancer Sci.

[137] Ding L, Getz G, Wheeler DA, Mardis ER, McLellan MD, Cibulskis K, Sougnez C, Greulich H, Muzny DM, Morgan MB, Fulton L, Fulton RS, Zhang Q (2008). Somatic mutations affect key pathways in lung adenocarcinoma. Nature.

[138] Cancer Genome Atlas Research N (2012). Comprehensive genomic characterization of squamous cell lung cancers. Nature.

[139] Lee E-S, Son D-S, Kim S-H, Lee J, Jo J, Han J, Kim H, Lee HJ, Choi HY, Jung Y, Park M, Lim YS, Kim K (2008). Prediction of recurrence-free survival in postoperative non-small cell lung cancer patients by using an integrated model of clinical information and gene expression. Clin Cancer Res.

[140] Buchholz M, Braun M, Heidenblut A, Kestler HA, Kloppel G, Schmiegel W, Hahn SA, Luttges J, Gress TM (2005). Transcriptome analysis of microdissected pancreatic intraepithelial neoplastic lesions. Oncogene.

[141] Anglesio MS, Arnold JM, George J, Tinker AV, Tothill R, Waddell N, Simms L, Locandro B, Fereday S, Traficante N, Russell P, Sharma R, Birrer MJ (2008). Mutation of ERBB2 provides a novel alternative mechanism for the ubiquitous activation of RAS-MAPK in ovarian serous low malignant potential tumors. Molecular cancer research.

[142] Tothill RW, Tinker AV, George J, Brown R, Fox SB, Lade S, Johnson DS, Trivett MK, Etemadmoghadam D, Locandro B, Traficante N, Fereday S, Hung JA (2008). Novel molecular subtypes of serous and endometrioid ovarian cancer linked to clinical outcome. Clin Cancer Res.

[143] Zhao H, Ljungberg B, Grankvist K, Rasmuson T, Tibshirani R, Brooks JD (2006). Gene expression profiling predicts survival in conventional renal cell carcinoma. PLoS medicine.

[144] Aran D, Sirota M, Butte AJ (2015). Systematic pan-cancer analysis of tumour purity. Nat Commun.

[145] Letunic I, Doerks T, Bork P (2015). SMART: recent updates, new developments and status in 2015. Nucleic Acids Res.

[146] Schultz J, Milpetz F, Bork P, Ponting CP (1998). SMART, a simple modular architecture research tool: identification of signaling domains. Proc Natl Acad Sci USA.

[147] Kieffer B, Driscoll PC, Campbell ID, Willis AC, van der Merwe PA, Davis SJ (1994). Three-dimensional solution structure of the extracellular region of the complement regulatory protein CD59, a new cell-surface protein domain related to snake venom neurotoxins. Biochemistry.

[148] Choi SH, Kong HK, Park SY, Park JH (2009). Metastatic effect of LY-6K gene in breast cancer cells. Int J Oncol.

[149] Tirosh Y, Ofer D, Eliyahu T, Linial M (2013). Short toxin-like proteins attack the defense line of innate immunity. Toxins (Basel).

[150] Kong HK, Yoon S, Park JH (2012). The regulatory mechanism of the LY6K gene expression in human breast cancer cells. J Biol Chem.

[151] Rhodes DR, Yu J, Shanker K, Deshpande N, Varambally R, Ghosh D, Barrette T, Pandey A, Chinnaiyan AM (2004). ONCOMINE: a cancer microarray database and integrated data-mining platform. Neoplasia.

[152] Oncomine. Compendia Bioscience, Inc. (2011). http://www.oncomine.org.

[153] Gao J, Aksoy BA, Dogrusoz U, Dresdner G, Gross B, Sumer SO, Sun Y, Jacobsen A, Sinha R, Larsson E, Cerami E, Sander C, Schultz N (2013). Integrative Analysis of Complex Cancer Genomics and Clinical Profiles Using the cBioPortal. Sci Signal.

[154] Cerami E, Gao J, Dogrusoz U, Gross BE, Sumer SO, Aksoy BA (2012). The cBio Cancer Genomics Portal: An Open Platform for Exploring Multidimensional Cancer Genomics Data (vol 2, pg 401, 2012). Cancer Discovery.

[155] Nikitin A, Egorov S, Daraselia N, Mazo I (2003). Pathway studio - the analysis and navigation of molecular networks. Bioinformatics.

[156] Győrffy B, Surowiak P, Budczies J, Lánczky A (2013). Online survival analysis software to assess the prognostic value of biomarkers using transcriptomic data in non-small-cell lung cancer. Plos One.

[157] Mizuno H, Kitada K, Nakai K, Sarai A (2009). PrognoScan: a new database for meta-analysis of the prognostic value of genes. BMC Med Genomics.

[158] UniProt Consortium (2015). UniProt: a hub for protein information. Nucleic Acids Res.

[159] Sievers F, Wilm A, Dineen D, Gibson TJ, Karplus K, Li W, Lopez R, McWilliam H, Remmert M, Söding J, Thompson JD, Higgins DG (2011). Fast, scalable generation of high-quality protein multiple sequence alignments using Clustal Omega. Mol Syst Biol.

[160] Waterhouse AM, Procter JB, Martin DMA, Clamp M, Barton GJ (2009). Jalview Version 2--a multiple sequence alignment editor and analysis workbench. Bioinformatics.

