# Supplementary material for: Distinct lymphocyte antigens 6 (Ly6) family members Ly6D, Ly6E, Ly6K and Ly6H drive tumorigenesis and clinical outcome
Source: Oncotarget. 2016 Feb 3;7(10):11165–93. doi: 10.18632/oncotarget.7163 (PMC4905465; doi:10.18632/oncotarget.7163)
Supplement: Supplementary file 1 [file oncotarget-07-11165-s001.pdf]

SUPPLEMENTARY TABLES

Supplementary Table S1: High Ly6D mRNA expression and its effect on survival outcome in multiple cancer types. High Ly6D expression was significantly correlated with poor clinical outcome in breast, colorectal, lung, gastric and ovarian cancer observed by KM plotter and PROGgeneV2.

See Supplementary File 1

Supplementary Table S2: High Ly6E mRNA expression and its effect on survival outcome in multiple cancers. High Ly6E expression was significantly correlated with poor clinical outcome in breast, colorectal, lung, gastric and ovarian cancer observed by KM plotter and PROGgeneV2.

See Supplementary File 1

Supplementary Table S3: High Ly6H mRNA expression and its effect on survival outcome in multiple cancers. High Ly6H expression was significantly correlated with poor clinical outcome in colorectal, lung, ovarian cancer and gastric cancer observed by KM plotter and PROGgeneV2.

See Supplementary File 1

Supplementary Table S4: High Ly6K mRNA expression and its effect on survival outcome in multiple cancer types. High Ly6K expression was significantly correlated with poor clinical outcome in breast, lung, ovarian and colorectal cancer observed by KM plotter and PROGgeneV2.

See Supplementary File 1
