# Supplementary material for: Distinct lymphocyte antigens 6 (Ly6) family members Ly6D, Ly6E, Ly6K and Ly6H drive tumorigenesis and clinical outcome
Source: Oncotarget. 2016 Feb 3;7(10):11165–93. doi: 10.18632/oncotarget.7163 (PMC4905465; doi:10.18632/oncotarget.7163)
Supplement: Supplementary file 2 [file oncotarget-07-11165-s002.pdf]

Supplementary Table 1

Type of Cancer	Database	Expression level: number of sample	Survival analysis	Restriction	Hazard Ratio	P-value
**Breast**	KM plotter	Low:818<br>High:790	Distance metastasis free	None	1.29	1.2E-02
		Low:231<br>High:120	Post progression free	None	1.57	9.0E-04
		Low:1133<br>High:2421	Relapse free	None	1.3	2.0E-05
	PROGgeneV2 (GSE19615)	Low:57<br>High:57	Relapse free	None	1.48	6.0E-03
**Colorectal**	PROGgeneV2 (GSE39582)	Low:25<br>High:26	Relapse free	None	1.19	4.7E-02
	PROGgeneV2 (GSE17537)	Low:25<br>High:26	Overall	None	1.63	2.0E-02
**Lung**	KM plotter	Low:712<br>High:270	First progression	None	1.33	6.0E-03
		Low:345<br>High:116	First progression	Adeno	1.71	1.0E-03
		Low:1323<br>High:603	Overall	None	1.49	1.7E-09
		Low:538<br>High:181	Overall	Adeno	2.11	7.6E-10
		Low:257<br>High:87	Post progression free	Adeno	1.48	6.0E-03
	PROGgeneV2 (GSE31210)	Low:112<br>High:113	Relapse free	Adeno	1.38	4.0E-04
	PROGgeneV2 (GSE26939)	Low:57<br>High:57	Overall	None	1.16	2.0E-03
	PROGgeneV2 (GSE41271)	Low:138<br>High:138	Overall	None	1.11	1.3E-02
**Gastric**	KM plotter	Low:209<br>High:432	Post progression free	None	1.38	4.7E-03
**Ovarian**	KM plotter	Low:517<br>High:190	Post progression free	None	1.22	4.9E-02

Supplementary Table 1: High Ly6D mRNA expression and its effect on survival outcome in multiple cancer types. High Ly6D expression was significantly correlated with poor clinical outcome in breast, colorectal, lung, gastric and ovarian cancer observed by KM plotter and PROGgeneV2.

Supplementary Table 2

Type of cancer	Database	Expression level: number of sample	Survival analysis	Restriction	Hazard Ratio	P-value
**Glioma**	PROGgeneV2 (GSE4412)	Low:14<br>High:14	Overall	None	2.34	2.6E-03
**Breast**	KM plotter	Low:75<br>High:60	Overall	Grade1	3.48	2.2E-02
		Low:646<br>High:653	Overall	None	1.4	7.0E-03
		Low:179<br>High:129	Relapse free	Grade1	2.52	1.0E-03
		Low:388<br>High:336	Relapse free	Grade2	1.53	1.0E-03
		Low:1113<br>High:2441	Relapse free	None	1.32	1.5E-05
		Low:479<br>High:1130	Distance metastasis free	None	1.71	1.9E-05
		Low:99<br>High:252	Post progression free	None	1.43	1.8E-02
	PROGgeneV2 (NKI)	Low:112<br>High:113	distance metastasis free	ER positive	1.44	0.0E+00
		Low:112<br>High:113	Overall	ER positive	1.39	2.0E-03
	PROGgeneV2 (GSE3494)	Low:15<br>High:16	Overall	None	2.73	2.0E-02
		Low:28<br>High:29	Overall	PgR negative	1.82	2.7E-02
**Gastric**	KM plotter	Low:314<br>High:562	Overall	None	2.08	1.3E-14
**Lung**	PROGgeneV2 (GSE31210)	Low:27<br>High:27	Relapse free	Stage 1b	1.7	4.6E-02
	PROGgeneV2 (GSE50081)	Low:22<br>High:23	Relapse free	Stage IIb	1.97	2.9E-02
	PROGgeneV2 (TCGA_LUAD)	Low:14<br>High:14	Overall	Stage IIIa	6.82	2.7E-02
**Ovarian**	KM plotter	Low:52<br>High:51	Overall	Stage IV, Serous Grade3	1.70	3.6E-02
**Colorectal**	PROGgeneV2 (GSE14333)	Low:93<br>High:94	Relapse free	None	1.77	5.0E-03
		Low:82<br>High:83	Relapse free	Age- >50 years	1.70	1.8E-02

Supplementary Table 2: High Ly6E mRNA expression and its effect on survival outcome in multiple cancers. High Ly6E expression was significantly correlated with poor clinical outcome in breast, colorectal, lung, gastric and ovarian cancer observed by KM plotter and PROGgeneV2.

Supplementary Table 3

Type of Cancer	Database	Expression level: number of sample	Survival analysis	Restriction	Hazard Ratio	P-value
**Colorectal**	PROGgeneV2 (GSE17536)	Low:70<br>High:75	Relapse free	None	7.60	3.3E-02
**Lung**	KM plotter	Low:557<br>High:425	First progression free	None	1.77	2.9E-09
		Low:87<br>High:257	Post progression free	None	1.47	1.5E-02
		Low:484<br>High:1442	Overall	None	1.30	6.0E-04
**Ovarian**	KM plotter	Low:305<br>High:506	Post progression free	None	1.28	1.6E-02
	PROGgeneV2 (GSE49997)	Low:96<br>High:97	Overall	None	1.34	3.4E-02
**Gastric**	KM plotter	Low:377<br>High:499	Overall	None	1.56	6.0E-07
		Low:336<br>High:499	First progression free	None	1.50	6.9E-05

Supplementary Table 3: High Ly6H mRNA expression and its effect on survival outcome in multiple cancers. High Ly6H expression was significantly correlated with poor clinical outcome in colorectal, lung, ovarian cancer and gastric cancer observed by KM plotter and PROGgeneV2.

Supplementary Table 4

Type of Cancer	Study	Expression status: number of sample	Survival analysis	Restriction	Hazard Ratio	P-value
**Breast**	PROGgeneV2 (GSE42568)	Low:51	Overall	None	1.25	2.1E-02
		High:52				
**Lung**	PROGgeneV2 (GSE41271)	Low:18	Relapse free	Stage IIb	2.02	2.0E-03
		High:21				
	PROGgeneV2 (GSE41271)	Low:17	Overall	Stage IIIb	1.57	1.3E-02
		High:18				
	PROGgeneV2 (GSE41271)	Low:18	Overall	Stage IIb	1.98	2.0E-03
		High:21				
**Ovarian**	PROGgeneV2 (GSE49997)	Low:96	Overall	None	1.30	8.0E-03
		High:97				
**Colorectal**	PROGgeneV2 (GSE28814)	Low:31	Relapse free	Stage 2	216.51	2.7E-03
		High:31				
		Low:31	Metastasis free	Stage 2	185.70	9.4E-03
		High:31				

Supplementary Table 4: High Ly6K mRNA expression and its effect on survival outcome in multiple cancer types. High Ly6K expression was significantly correlated with poor clinical outcome in breast, lung, ovarian and colorectal cancer observed by KM plotter and PROGgeneV2.
